# Commercial exergames for rehabilitation of physical health and quality of life: a systematic review of randomized controlled trials with adults in unsupervised home environments

**DOI:** 10.3389/fpsyg.2023.1155569

**Published:** 2023-06-02

**Authors:** Marco Rüth, Mona Schmelzer, Kateryna Burtniak, Kai Kaspar

**Affiliations:** Department of Psychology, University of Cologne, Cologne, Germany

**Keywords:** commercial exergames, physical health, quality of life, rehabilitation, home-based exercise, unsupervised training, randomized controlled trials, systematic review

## Abstract

**Background:**

Commercial exergames are widely available tools that can support physical rehabilitation at home. However, the effects of the unsupervised use of commercial exergames in home environments are not yet clear. Hence, we provide a systematic review on the effects of unsupervised commercial exergaming at home on adults' physical health (RQ1) and quality of life (RQ2). We also scrutinize adults' experiences with exergaming at home regarding participant support, adherence, and adverse outcomes (RQ3).

**Methods:**

We searched Web of Science, PsycINFO, PubMed, Embase, and CINAHL for peer-reviewed randomized controlled trials with adults in need of rehabilitation. Overall, 20 studies (1,558 participants, 1,368 analyzed) met our inclusion criteria. The quality of evidence was assessed with the Cochrane risk of bias tool.

**Results:**

Effects of unsupervised commercial exergaming at home on physical health were higher in seven studies and similar in five studies regarding the respective comparison or control conditions; eight studies reported non-significant findings. Of the 15 studies that also examined effects on quality of life, improvements were higher in seven studies and similar in two studies regarding the respective comparison or control conditions; results were non-significant in six studies. Participant support consisted of setup of the exergaming system, instructions, training, and contact with participants. Adherence was high in eight studies, moderate in six studies, and low in one study. Adverse outcomes related to exergaming were found in four studies and were at most moderate. Concerning the quality of evidence, six studies were related to a high risk of bias due to outcome reporting bias or ceiling effects in the primary outcome. Additionally, 10 studies yielded some concerns, and four studies were related to a low risk of bias.

**Discussion:**

This systematic review summarizes promising evidence that the unsupervised use of commercial exergames can support and complement rehabilitation measures in home environments. Still, future studies based on larger samples and using more recent commercial exergames are needed to obtain more high-quality evidence on the effects of different exercise prescriptions. Overall, considering the necessary precautions, the unsupervised use of commercial exergames at home can improve the physical health and quality of life in adults with needs for physical rehabilitation.

**Systematic review registration:**

https://www.crd.york.ac.uk/prospero/display_record.php?ID=CRD42022341189, identifier: PROSPERO, Registration number: CRD42022341189.

## 1. Introduction

Rehabilitation is an important cornerstone for people to restore and improve their physical health and quality of life (WHO, 2021). Rehabilitation encompasses “a set of interventions designed to optimize functioning and reduce disability in individuals with health conditions in interaction with their environment” (WHO, 2021, p. 1). Accordingly, physical health refers to the wellbeing and functioning of body parts that are relevant for physical interactions with the environment, such as limbs and muscles for grasping, walking, and other forms of physical activity. In addition, the term quality of life comprises the overall wellbeing and health of people (cf. Fayers and Machin, 2015), including health-related and psychological measures as well as rehabilitation benefits that go beyond physical health. It has been shown that an adequate amount of physical activity is key for people's health at every age, yet about 80% of adolescents and almost 30% of adults worldwide are not sufficiently physically active (Guthold et al., 2018, 2020; WHO, 2022). Relatedly, rehabilitation measures are not only important for *patients* with long-term or severe physical impairments but could support about every third *person* worldwide to improve their health condition (Cieza et al., 2020). In this regard, several studies have shown that exergaming can effectively support rehabilitation in terms of physical health and quality of life (e.g., Elena et al., 2021; Shida et al., 2021; Blasco-Peris et al., 2022; Gelineau et al., 2022). Exergaming refers to the use of video games that require players to be physically active, such as strength and balance exercises, and involves physical, cognitive, and psychological processes. Accordingly, exergaming has been related to several physical, psychological, and educational effects based on theories of motor learning, social cognitive theory, and self-determination theory (e.g., Peeters et al., 2013; Rüth and Kaspar, 2021). However, some exergames are customized to characteristics of people with specific symptoms or a certain pathology and are not widely available (Schättin et al., 2021). In contrast, commercial exergames are widely available tools that could support physical rehabilitation in terms of more general physical health and quality of life.

Commercial exergames refer to exergames that can be played on commercially available devices that can be purchased by the general public. Commercial exergames have been used in supervised contexts, such as rehabilitation centers (e.g., Prosperini et al., 2021) and schools (e.g., Rüth and Kaspar, 2020). However, supervised exergaming requires personal and financial resources such as rehabilitation professionals, which are particularly lacking in middle- and low-income countries (WHO, 2021). In addition, rehabilitation measures do not necessarily improve more in supervised vs. unsupervised settings (Lilios et al., 2021). In fact, meta-analytic findings indicate that improvements can be even higher in home environments than in supervised environments (Cugusi et al., 2021; Prosperini et al., 2021). Relatedly, meta-analytic findings indicate that self-rehabilitation programs can be as effective as conventional therapy regarding motor outcomes of adults who have had a stroke (Everard et al., 2021). Moreover, compared to center-based rehabilitation and telerehabilitation, exergaming at home can save healthcare expenses (Klompstra et al., 2022) as well as travel costs and time by allowing people to stay in their familiar home environment. Thus, the unsupervised use of commercial exergames at home could be a powerful tool to support rehabilitation, yet a systematic review on the effects on physical health and quality of life is still missing.

## 2. Using commercial exergames to improve physical health and quality of life

The use of commercial exergames has been related to several benefits for physical health and quality of life. For instance, a meta-analysis showed that the use of commercial exergames had a moderate positive effect on balance in adults with neurological pathologies (Prosperini et al., 2021). This effect was slightly higher in home environments (Hedge's *g* = 0.52) than in supervised environments (*g* = 0.41). Concerning arm, hand, and leg rehabilitation, meta-analytic findings indicate that playing commercial exergames at home has beneficial effects on adults with neurological diseases that are at least comparable with conventional therapy or usual care (Perrochon et al., 2019). Compared to conventional stroke rehabilitation, playing commercial exergames can alleviate motor impairment and improve motor function, according to meta-analytic results (Unibaso-Markaida and Iraurgi, 2021). Another meta-analysis on the effects of unsupervised exergaming at home and supervised exergaming in health facilities on adults with chronic diseases found that playing commercial exergames can have larger effects than conventional care on several facets of quality of life such as physical and social functioning (Cugusi et al., 2021). Irrespective of the pathology, the use of commercial exergames at home can have several positive effects on physical, cognitive, and psychological outcomes (e.g., Bonnechère et al., 2016; Rüth and Kaspar, 2021). However, the effects of the unsupervised use of commercial exergames in home environments on physical health and quality of life across pathologies are not yet clear. Hence, we provide a systematic overview of available evidence on the effects of the unsupervised use of commercial exergames at home on adults' physical health (RQ1) and quality of life (RQ2). In addition, different methods can be used to ensure and measure compliance with exergaming interventions in home environments (Donoso Brown et al., 2020), and in few cases exergaming has been related to adverse outcomes, such as musculoskeletal disorders, accidental falls, increased spasticity, or dizziness (Prosperini et al., 2021). Hence, specifically concerning unsupervised exergaming, information is needed on adults' autonomy, compliance, and safety regarding the rehabilitation measures. Thus, we also scrutinize how adults experience the unsupervised use of commercial exergames at home in terms of participant support (RQ3a), adherence (RQ3b), and adverse outcomes (RQ3c).

## 3. Methods

This systematic review follows the Preferred Reporting Items of Systematic Reviews and Meta-Analyses (PRISMA) guidelines (Page et al., 2021) and has been registered with the International Prospective Register of Systematic Reviews (PROSPERO, Registration number: CRD42022341189).

### 3.1. Database search strategy

**Figure 1:**
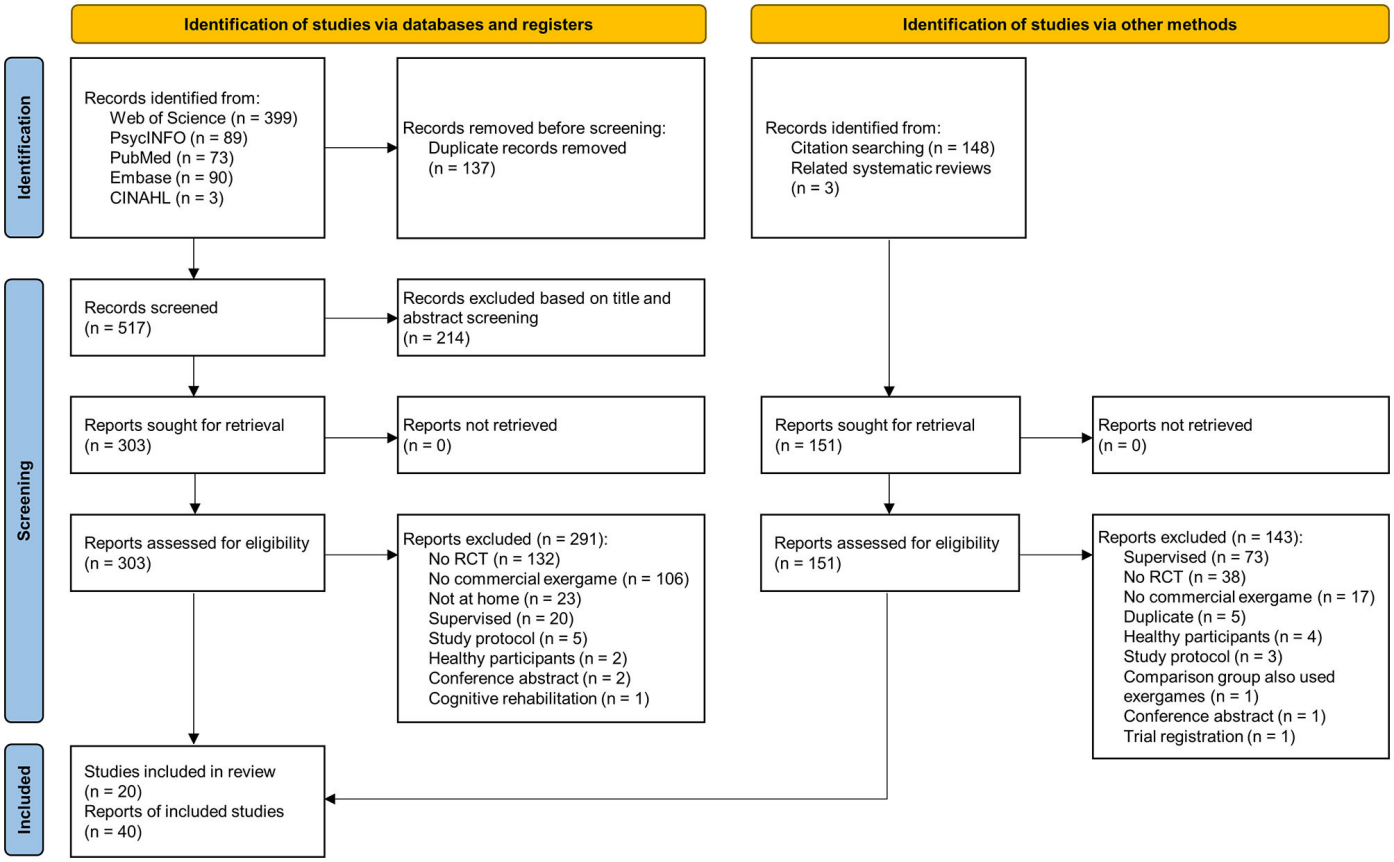
Study selection process based on the PRISMA 2020 statement.

The databases Web of Science Core Collection, PsycINFO (via EBSCO), and PubMed, Embase, and CINAHL (via CENTRAL) were searched without time restrictions. Each database was searched first on July 12, 2022. In addition, we updated our search results from each database last on January 30, 2023. The search strings are provided in Supplementary Table S1. In addition, we checked reports included in related systematic reviews as well as all citing and cited references of eligible studies. Figure 1 provides an overview of the study selection process.

### 3.2. Eligibility criteria and study selection process

Following the PICOS (population, intervention, comparison, outcome, study) statement, the inclusion and exclusion criteria of our systematic review can be found in Table 1. We included peer-reviewed randomized controlled trials (RCTs) published in English language that evaluated the effects of the unsupervised use of commercial exergames at home on the physical health or quality of life in adults in need of rehabilitation. The comparison was anything but unsupervised exergaming at home (e.g., conventional rehabilitation, continuing usual activities, etc.). Physical health outcomes include, inter alia, results from questionnaires related to physical health as well as from tests related to physical activity, such as walking distance and gait. Outcomes related to quality of life include, inter alia, health-related quality of life, confidence in physical activities, fatigue, anxiety, and perceived pain.

**Table 1:** Inclusion and exclusion criteria in terms of population, intervention, comparator, outcome, and study type (PICOS).

**PICOS component**	**Selection criteria**	
Population	Inclusion:	Adults (age ≥ 18 years) enrolled in physical rehabilitation programs and living in their own homes or home-like settings (retirement homes/communities, nursing homes, or assisted living homes).
	Exclusion:	Children and adolescents (age < 18 years); healthy adults; adults enrolled in cognitive rehabilitation; adults living in medical facilities and medical care units.
Intervention	Inclusion:	Fully unsupervised use of commercial exergames in terms of video games that require physical exertion and that can be played on commercially available devices that can be purchased by the general public; unsupervised use of commercial exergames after a phase of supervised use of commercial exergames.
	Exclusion:	Use of games that do not require physical exertion; fully supervised use of exergames; use of exergames that cannot be played on commercially available devices that can be purchased by the general public.
Comparison	Inclusion:	Anything but unsupervised exergaming at home (e.g., conventional rehabilitation, continuing usual activities, etc.).
Outcome	Inclusion:	Quantitative measures of physical health (e.g., limb function, disease activity), including physical activity (e.g., balance, walking). Quantitative measures of quality of life (e.g., confidence in physical activity, anxiety, and perceived pain). Quantitative and qualitative measures of experiences with the intervention (e.g., adherence, adverse outcomes, enjoyment/fun).
Study	Inclusion:	Randomized controlled trials including original research studies and pilot/feasibility studies.
	Exclusion:	Quasi-experimental studies, solely qualitative studies, case studies, study protocols, theoretical articles, reviews, and conference abstracts.
Date		No restrictions.
Language		English.

Titles and abstracts of eligible studies were screened and coded independently by three reviewers (MR, MS, KB). For all included studies, we performed data extraction and synthesis, where possible. If necessary, the authors of the included articles were contacted to request missing or additional data. The quality of evidence was assessed using the revised tool to assess the risk of bias in randomized trials (RoB 2) (Sterne et al., 2019). Following this approach, studies are rated in five domains and may be related to a low risk of bias, some concerns, or a high risk of bias, whereas “the overall risk of bias generally corresponds to the worst risk of bias in any of the domains” (Sterne et al., 2019, p. 5). Risk of bias assessments are used to assess the robustness of the reported results and should not be misinterpreted as an evaluation of the overall quality of the studies. Three reviewers (MR, MS, KB) inspected the final study reports and study protocols when available. Disagreements were resolved via discussion until a consensus was reached. To assess outcome reporting bias, we compared statistical analysis plans and outcomes pre-specified in protocols and study registers with analyses and results in the final study reports. We also checked if the measures mentioned in the methods section were included in the results section. Finally, we addressed non-reporting bias by searching for study reports of study protocols that met our inclusion criteria.

### 3.3. Data extraction

Based on the PICOS statement and the template for intervention description and replication (TIDieR) checklist, three reviewers (MR, MS, KB) extracted essential information from eligible studies (cf. Hoffmann et al., 2014; Higgins et al., 2019). We provide information (1) on the report: author(s), year of publication, and study location(s); (2) on the study design: RCT type and characteristics, including blinding and randomization procedures; (3) on the participants: participant type (pathology, diagnostic criteria) as well as characteristics of the baseline and final/analyzed sample (recruitment, sample size, age, and gender); (4) on the intervention: background and aim, devices/exergames used, intended protocol, and participant support; (5) on the realization of the control/comparison group(s); (6) on the outcome definition and measurement: physical health, quality of life, and experiences with the intervention (adherence and adverse outcomes); and (7) on the main findings for physical health, quality of life, and experiences with the intervention (adherence and adverse outcomes). All items from the TIDieR checklist were considered as follows: why (background and aim), what (devices/exergames used, intended protocol, and participant support), who provided (participant support), how, where, when, how much, tailoring, modifications (intended protocol), and how well (intended protocol and adherence). Specifically, we extracted information on the following general exercise and training variables that are relevant to exercise prescription (cf. Burnet et al., 2019; Herold et al., 2019): frequency, intensity, time, type, density, duration, and enjoyment. Relatedly, we also examined whether the studies considered the following general training principles: variation, specificity, overload, progression, reversibility, and periodization and programming (Herold et al., 2019). In addition, we extracted information on financial support and financial conflicts of interest of study authors. A meta-analysis could not be undertaken due to the heterogeneity of sample characteristics, exergaming interventions, and outcome measures.

## 4. Results

### 4.1. Study selection

Database searching resulted in 654 records. After the removal of duplicates, 517 articles were independently screened. In addition, 151 records were identified through citation searching and screening of related systematic reviews. Overall, 20 studies and 40 reports were included in this systematic review, including final reports, trial protocols, trial registry records, and secondary analyses (see Figure 1).

### 4.2. Characteristics of included studies

In the following sections, we present key characteristics of the included studies, including (1) location, design, and participants, (2) background and aims of the studies, (3) exergaming interventions and control/comparison groups, (4) effects of exergaming on physical health, (5) effects of exergaming on quality of life, (6) experiences with the exergaming interventions, and (7) financial support and financial conflict of interest. More specific information about the exergaming interventions and control/comparison groups regarding exercise and training variables can be found in Table 2. Table 3 provides a concise overview of the effects of unsupervised exergaming on physical health (RQ1) and quality of life (RQ2) as well as on adults' experiences with unsupervised exergaming at home in terms of participant support (RQ3a), adherence (RQ3b), and adverse outcomes (RQ3c). Additionally, a comprehensive overview of the details of each study can be found in Supplementary Table S2.

**Table 2:** Exercise and training variables in the intervention and control/comparison groups.

**Study**	**Exercise variables (relevant in an exercise session)**			**Training variables (relevant in a training program)**				**Findings**	
**References and pathology**	**Type**	**Intensity**	**Session duration**	**Frequency**	**Density**	**Program duration**	**Enjoyment**	**Physical health**	**Quality of life**
Adie et al. (2017) Arm weaknesses following a stroke	IG: 4 Wii Sports games (bowling, tennis, golf, baseball) CG: tailored arm exercises IG and CG: usual care and rehabilitation	NI	≤45 min (+15 min warm-up exercises)	7 days/week	NI	6 weeks	NI	= (arm function)	= (health state)
Ambrosino et al. (2020) Rheumatoid arthritis	IG: 5 Wii Fit games (running, skiing, balloons shooting, bike slalom, balls moving through labyrinth) CG: usual activities	IG: NI CG: N/A	IG: 50 min (10 min/game) CG: N/A	IG: 7 days/week CG: N/A	IG: NI CG: N/A	8 weeks	NI	= (global health)	+ (difficulty with activities) – (fatigue)
Golla et al. (2018) Stroke	IG: 4 Wii Fit Plus balance games (ski slalom, table tilt, penguin slide, balance bubble) CG: conventional balance exercises	NI	30 min	≥3 times/week	NI	6 weeks	NI	n.s. (balance, gait)	+ (balance confidence)
Imam et al. (2017) Lower limb amputation	IG: 4 Wii Fit games (yoga, balance games, strength training, aerobics) CG: Wii Big Brain Academy Degree program (cognitive tasks)	IG: NI CG: N/A	40 min	3 days/week	1–2 rest days^a^	4 weeks	NI	+ (walking capacity) n.s. (physical activity, steps per day, cognitive-motor interaction)	n.s. (balance confidence)
Jaarsma et al. (2021a) Heart failure	IG: 5 Wii Sports games (baseball, bowling, boxing, golf, tennis) CG: protocol-based physical activity advice (motivational support)	IG: NI CG: N/A	IG: 30 min CG: N/A	IG: 5 days/week CG: N/A	IG: NI CG: N/A	12 weeks	NI	+ (muscle function) n.s. (walking capacity, physical activity)	n.s. (exercise motivation, exercise self-efficacy)
Meldrum et al. (2015) Unilateral peripheral vestibular loss	IG: 5 Wii Fit Plus games representing balance exercises (yoga, leg exercises, balance games, aerobics, training plus games) CG: conventional balance exercises using a foam balance mat IG and CG: similar gaze stabilization exercises and a graded walking program	NI	15 min	5 days/week	NI	6 weeks	IG: more enjoyment than CG CG: NI	= (gait speed, standing balance)	= (balance confidence, anxiety, depression, rehabilitation benefits)
Prosperini et al. (2013) Multiple sclerosis	IG: 7 Wii Fit Plus balance games (zazen, table tilt, ski slalom, penguin slide, tightrope walk, soccer heading, balance bubble) CG: usual activities	IG: NI CG: N/A	IG: 30 min (10 min/game) CG: N/A	IG: 7 days/week CG: N/A	IG: 1 rest day/week was allowed CG: N/A	12 weeks	NI	+ (walking speed, static and dynamic balance)	+ (lower physical and psychological impact of multiple sclerosis)
Punt et al. (2016, 2017) Ankle sprain	IG: 4 Wii Fit balance games (ski slalom, table tilt, penguin slide, balance bubble) CG1: conventional physical therapy and advice to practice at home CG2: no exercise therapy	IG: preferred difficulty level CG1: difficulty level adjusted to progress CG2: N/A	IG: ≥30 min CG1: 30 min CG2: N/A	IG: 2 times/week CG2: 9 sessions over 6 weeks CG2: N/A	IG and CG1: NI CG2: N/A	6 weeks	NI	= (foot and ankle ability, gait speed, cadence, step length) + (single-support time)	+ (pain during rest) = (pain during walking)
Sajid et al. (2016) Prostate cancer	IG: Wii Fit games (exact games not specified) CG1: progressive home-based aerobic walking exercise program and therapeutic resistance band exercise program CG2: usual care	IG: similar intensity as in CG1 CG1: low to moderate^b^ CG2: N/A	IG and CG1: similar (session duration unknown) CG2: N/A	IG and CG1: ≥5 days/week CG2: N/A	IG and CG1: NI CG2: N/A	6 weeks	NI	n.s.^c^ (physical performance, steps per day, handgrip strength, lean muscle mass, chest press repetitions)	N/A
Sanders et al. (2020) Stroke affecting the hand	IG: hand exercises with MusicGlove CG: conventional hand therapy exercises depicted in a booklet	NI	≥3 h/week (session duration unknown)	NI	NI	3 weeks	NI	n.s. (gripping function)	N/A
Sanders et al. (2022) Spinal cord injury affecting hand function	IG: hand exercises with MusicGlove CG: 18 conventional hand therapy exercises	NI	IG: ≥3 h/week (session duration unknown) CG: ≥3 h/week (1 h/session)	≥3 times/week	IG: NI CG: 2 sets of repetitions, 2 times/day	3 weeks	NI	n.s. (gripping function, sensorimotor hand function)	N/A
Tao et al. (2022) Lower limb amputation	IG: 4 Wii Fit games (yoga, balance games, strength training, aerobics) CG: Wii Big Brain Academy Degree program (cognitive tasks)	NI	As much as participants liked (session duration unknown)	As much as participants liked (frequency unknown)	NI	4 weeks	NI	n.s. (walking capacity, lower limb, dynamic standing balance)	+ (balance confidence)
Tefertiller et al. (2019) Traumatic brain injury	IG: 6 Xbox Kinect Adventures and Xbox Kinect Sports games (20,000 leaks, soccer, table tennis, rallyball, beach volleyball, river rush)^d^ CG: traditional home-based exercise program^d^	NI	30 min	3–4 times/week	NI	12 weeks	NI	= (balance)	n.s. (balance confidence, community participation)
Thomas et al. (2017) Multiple sclerosis	IG: Wii Sports games, Wii Sports Resort games, and Wii Fit Plus games (exact games not specified) CG: usual care	NI	NI	NI	NI	IG: 12 months CG: 6 months (delayed group) IG and CG (after delay): first 3 weeks were super-vised	IG: most participants enjoyed the exergaming intervention CG: NI	n.s. (physical activity, self-efficacy, balance, gait)	n.s. (self-efficacy, hospital depression, hospital anxiety, psychological impact of multiple sclerosis on day-to-day life)
Villumsen et al. (2019) Prostate cancer	IG: 3 Xbox Kinect 360 games (Adventures, Sports, and Your Shape Fitness Evolved 2012) CG: continuation of normal daily activities	IG: at own convenience CG: N/A	IG: 60 min CG: N/A	IG: 3 times/week CG: N/A	IG: NI CG: N/A	12 weeks	NI	+ (walking capacity)	n.s. (global health)
Yacoby et al. (2019) Stroke	IG: 3–5 games, either using Xbox Kinect (standing), or PlayStation EyeToy (sitting) CG: self-administered Graded Repetitive Arm Supplementary Program (GRASP) and 3 lower extremity exercises (stretching, marching, and stepping)	IG: NI CG: 3 levels of exercises	60 min	6 times/week	NI	5 weeks (+4 optional weeks)	IG: high enjoyment, slightly higher than CG CG: moderate to high enjoyment	n.s. (upper extremity, perceived balance improvement)	N/A
Yuen et al. (2019) Idiopathic pulmonary fibrosis	IG: Wii Fit games (exact games not specified) with Wii U Balance Board CG: cognitive digital game on Wii U without Wii U Balance Board IG and CG: encouragement to engage in physical activity	IG: moderate to heavy^e^ CG: not physically taxing	IG: 30 min exergaming + 30 min physical activity CG: 30 gaming + 30 min physical activity	3 times/week gaming + 3 times/week physical activity	NI	12 weeks	NI	n.s. (walking capacity)	n.s. (health-related quality of life)
Zadro et al. (2019) Chronic low back pain	IG: 4 Wii Fit U games (yoga, muscle/strength training, aerobics, balance games) CG: continuation of usual activities (including care-seeking behaviors)	IG: moderate^f^ CG: N/A	IG: 60 min CG: N/A	IG: 3 times/week CG: N/A	IG: ≥1 day off CG: N/A	8 weeks	NI	+ (function^g^, engagement in physical activity)	+ (pain self-efficacy, pain intensity)
Zahedian-Nasab et al. (2021) Fall risk	IG: 4 Xbox Kinect sports pack games (ski, penalty, goalkeeper, darts) CG: routine programs of the nursing homes (jogging in the nursing home, table tennis, some artistic activities)	NI	IG: 30–60 min CG: NI	IG: 2 times/week CG: NI	NI	6 weeks	NI	+ (balance)	+ (fear of falling)
Zondervan et al. (2016) Chronic stroke	IG: hand exercises with MusicGlove CG: conventional hand therapy exercises depicted in a booklet	NI	≥3 h/week (session duration unknown)	≥3 times/week	NI	3 weeks	NI	= (gripping function) + (motor activity)	N/A

CG, Control/Comparison group; IG, Intervention Group; N/A, Not Applicable; NI, No Information; PH, Physical Health; QoL, Quality of Life. For studies that include supervised and unsupervised uses of exergames, data only refer to the unsupervised phase.

+, Significantly higher increase in the exergaming/intervention group in relation to the control/comparison group(s).

=, Similar increase in the exergaming/intervention group and the control/comparison group(s).

n.s., No significant change in the exergaming/intervention group and the control/comparison group(s).

^a^Exercising was on Mondays, Wednesdays, and Fridays; rest days were Tuesdays, Thursdays, and the weekends.

^b^Low to moderate for resistance exercises (perceived exertion of 3–5 on the American College of Sports Medicine revised rating scale) or moderate for aerobic walking exercise program.

^c^Exergaming/intervention group in relation to the control group (three-group design).

^d^Focus of the games vs. exercises was based on the most impaired subscale of the Balance Evaluation Systems Test (BESTest).

^e^Based on perceived dyspnea level.

^f^Rating of 13 on the Borg rating scale.

^g^But not in case of family history of activity-limiting lower back pain (see Zadro et al., 2020).

**Table 3:** Overview of the empirical outcomes of the included studies.

**References**	**RQ1 (physical health)**	**RQ2 (quality of life)**	**RQ3a (participant support)**	**RQ3b (adherence)**	**RQ3c (adverse outcomes)**
Adie et al. (2017)	=	=	Yes^1, 2, 3, 4^	High	No
Ambrosino et al. (2020)	=	+^a^	Yes^3, 4^	High	No
Golla et al. (2018)	n.s.	+	Yes^1, 2, 3, 4^	High	No
Imam et al. (2017)	+^a^	n.s.	Yes^3, 4^	High	No
Jaarsma et al. (2021a)	+^a^	n.s.	Yes^1, 2, 3, 4^	Moderate	No
Meldrum et al. (2015)	=	=	Yes^2, 3, 4^	High	Yes
Prosperini et al. (2013)	+	+	Yes^1, 3, 4^	High	Yes
Punt et al. (2016, 2017)	=^b^	+	Yes^2, 3^	N/A	N/A
Sajid et al. (2016)	n.s.^c^	N/A	Yes^2, 3, 4^	N/A	N/A
Sanders et al. (2020)	n.s.	N/A	Yes^1, 3^	Moderate	N/A
Sanders et al. (2022)	n.s.	N/A	Yes^1, 3^	Moderate	N/A
Tao et al. (2022)	n.s.	+	Yes^3^	Moderate	No
Tefertiller et al. (2019)	=	n.s.	Yes^1, 3^	Moderate	No
Thomas et al. (2017)	n.s.	n.s.	Yes^2, 3, 4^	N/A	Yes
Villumsen et al. (2019)	+	n.s.	Yes^2, 4^	High	Yes
Yacoby et al. (2019)	n.s.	N/A	Yes^1, 3, 4^	Moderate	No
Yuen et al. (2019)	n.s.	n.s.	Yes^4^	Low	No
Zadro et al. (2019)	+	+	Yes^1, 2, 4^	High	No
Zahedian-Nasab et al. (2021)	+	+	Yes^2, 4^	N/A	N/A
Zondervan et al. (2016)	+^a^	N/A	Yes^1, 3, 4^	N/A	N/A

N/A, Not Applicable. Adherence was categorized as high (> 70% of the intended goal), moderate (30–50%), or low (< 30%).

+, Significantly higher increase in the exergaming/intervention group in relation to the control/comparison group(s).

=, Similar increase in the exergaming/intervention group and the control/comparison group(s).

n.s., No significant change in the exergaming/intervention group and the control/comparison group(s).

^a^Based on one of several outcomes.

^b^Based on most outcomes.

^c^Exergaming/intervention group in relation to the control group (three-group design).

^1^Setup: participants were supported in setting up the exergaming system.

^2^Instructions: participants received exercise instructions.

^3^Training: participants received some training before the start of the unsupervised phase of the exergaming intervention.

^4^Contact: participants were contacted by professionals or could contact professionals during the unsupervised phase of the exergaming intervention.

#### 4.2.1. Location, design, and participants

The included studies were conducted in different countries. Six studies were conducted in the United States (Sajid et al., 2016; Zondervan et al., 2016; Tefertiller et al., 2019; Yuen et al., 2019; Sanders et al., 2020, 2022), two in Canada (Imam et al., 2017; Tao et al., 2022), two in Italy (Prosperini et al., 2013; Ambrosino et al., 2020), two in the United Kingdom (Adie et al., 2017; Thomas et al., 2017), one in Australia (Zadro et al., 2019), one in Denmark (Villumsen et al., 2019), one in Germany (Golla et al., 2018), one in Iran (Zahedian-Nasab et al., 2021), one in Ireland (Meldrum et al., 2015), one in Israel (Yacoby et al., 2019), one in Switzerland (Punt et al., 2016), and one in multiple countries (Jaarsma et al., 2021a).

Sixteen studies used parallel RCT designs (Meldrum et al., 2015; Punt et al., 2016; Sajid et al., 2016; Adie et al., 2017; Imam et al., 2017; Thomas et al., 2017; Golla et al., 2018; Tefertiller et al., 2019; Villumsen et al., 2019; Yacoby et al., 2019; Yuen et al., 2019; Zadro et al., 2019; Ambrosino et al., 2020; Jaarsma et al., 2021a; Zahedian-Nasab et al., 2021; Tao et al., 2022) and four crossover RCT designs (Prosperini et al., 2013; Zondervan et al., 2016; Sanders et al., 2020, 2022). More specifically, seven of the included studies were pilot studies (Prosperini et al., 2013; Sajid et al., 2016; Thomas et al., 2017; Golla et al., 2018; Yacoby et al., 2019; Yuen et al., 2019; Ambrosino et al., 2020), four were feasibility studies (Zondervan et al., 2016; Imam et al., 2017; Sanders et al., 2020, 2022), and two were multicenter studies (Adie et al., 2017; Jaarsma et al., 2021a). Moreover, two studies included an additional comparison group (i.e., three groups) (Punt et al., 2016; Sajid et al., 2016).

Overall, data were collected from 1,558 participants (1,368 analyzed), with final/analyzed sample sizes ranging from 10 participants (Sanders et al., 2022) to 464 participants (Jaarsma et al., 2021a). More specifically, nine studies included 30 participants or less (Sajid et al., 2016; Zondervan et al., 2016; Imam et al., 2017; Thomas et al., 2017; Golla et al., 2018; Yacoby et al., 2019; Yuen et al., 2019; Sanders et al., 2020, 2022), and two studies included more than 100 participants (Adie et al., 2017; Jaarsma et al., 2021a). Most participants were older adults, since participants in 10 studies had a mean age of above 60 years, including those studies with the highest sample sizes (Sajid et al., 2016; Adie et al., 2017; Imam et al., 2017; Golla et al., 2018; Villumsen et al., 2019; Yuen et al., 2019; Zadro et al., 2019; Jaarsma et al., 2021a; Zahedian-Nasab et al., 2021; Tao et al., 2022). Based on the available information on gender (*n* = 1,532), the studies include more male (63.77%; *n* = 977) than female participants (36.23%; *n* = 555).

#### 4.2.2. Background and aims of the studies

Regarding the background of the included studies, we noted that only four works contained explicit references to theoretical frameworks regarding their intervention (Imam et al., 2017; Thomas et al., 2017; Jaarsma et al., 2021b; Tao et al., 2022). Imam et al. (2017) and Tao et al. (2022) used a similar intervention that was based on social cognitive theory and aimed to address all four sources of self-efficacy. Jaarsma et al. (2021b) outlined a conceptual model on beneficial effects of exergaming on health behaviors, exercise capacity, and health via motivation, physical activity, and self-efficacy. Thomas et al. (2017) mentioned that their intervention considered social cognitive theory, cognitive behavioral theory, and self-determination theory. Notably, Sanders et al. (2022) did not mention that their intervention was based on a theory, but that participants could adjust the game for optimal challenges in line with motor learning theory. Most other studies were based on an empirical background in terms of previous meta-analytic results or empirical findings from individual studies (see Supplementary Table S2).

Concerning the aim of the exergaming interventions, commercial exergames were used for the rehabilitation of various pathologies. Stroke was addressed in five studies (Zondervan et al., 2016; Adie et al., 2017; Golla et al., 2018; Yacoby et al., 2019; Sanders et al., 2020), multiple sclerosis in two studies (Prosperini et al., 2013; Thomas et al., 2017), prostate cancer in two studies (Sajid et al., 2016; Villumsen et al., 2019), and lower limb amputation in two studies (Imam et al., 2017; Tao et al., 2022). One study each included participants with rheumatoid arthritis (Ambrosino et al., 2020), heart failure (Jaarsma et al., 2021a), unilateral peripheral vestibular loss (Meldrum et al., 2015), ankle sprain (Punt et al., 2016), spinal cord injury (Sanders et al., 2022), traumatic brain injury (Tefertiller et al., 2019), idiopathic pulmonary fibrosis (Yuen et al., 2019), chronic low back pain (Zadro et al., 2019), and fall risk (Zahedian-Nasab et al., 2021). An overview of the games used in the case of each pathology can be found in Table 2.

#### 4.2.3. Exergaming interventions and control/comparison groups

To facilitate comparisons between the procedures used in intervention and control/comparison groups, Table 2 provides a detailed overview of exercise variables relevant in an exercise session (type, intensity, and session duration) and training variables relevant in a training program (frequency, density, program duration, and enjoyment).

First, regarding the type of exercise, different exergames were used: 10 studies (50%) included only Wii Fit games (Prosperini et al., 2013; Meldrum et al., 2015; Punt et al., 2016; Sajid et al., 2016; Imam et al., 2017; Golla et al., 2018; Yuen et al., 2019; Zadro et al., 2019; Ambrosino et al., 2020; Tao et al., 2022), two studies (10%) only Wii Sports games (Adie et al., 2017; Jaarsma et al., 2021a), and one study (5%) used both Wii Fit and Wii Sports games (Thomas et al., 2017). Three studies (15%) used only Xbox Kinect games (Tefertiller et al., 2019; Villumsen et al., 2019; Zahedian-Nasab et al., 2021), and one study (5%) used Xbox Kinect games or PlayStation EyeToy games (Yacoby et al., 2019). Finally, three studies (15%) used a game that comes with the commercially available device MusicGlove (Zondervan et al., 2016; Sanders et al., 2020, 2022). Consequently, participants in these studies completed different types of activities, such as yoga (Wii Fit), baseball (Wii Sports), table tennis (Xbox), or gripping movements (MusicGlove). Moreover, participants in 13 studies (65%) could complete between four and seven activities using Wii Fit, Wii Sports, or Xbox games, participants in three studies (15%) played one game using the MusicGlove device, and the number of games was not specified in the remaining four studies (20%).

In the control/comparison groups, participants most frequently completed conventional exercises, which was the case in eight studies (40%) (Meldrum et al., 2015; Punt et al., 2016; Zondervan et al., 2016; Golla et al., 2018; Tefertiller et al., 2019; Yacoby et al., 2019; Sanders et al., 2020, 2022), followed by usual activities in four studies (20%) (Prosperini et al., 2013; Villumsen et al., 2019; Zadro et al., 2019; Ambrosino et al., 2020) and usual care in terms of conventional rehabilitation in two studies (10%) (Thomas et al., 2017; Zahedian-Nasab et al., 2021). In addition, three studies (15%) realized playing cognitive digital games (Imam et al., 2017; Yuen et al., 2019; Tao et al., 2022), two studies (10%) implemented tailored exercises (Sajid et al., 2016; Adie et al., 2017), and one study (5%) included physical activity advice as a comparison group (Jaarsma et al., 2021a).

Second, only three studies (15%) specified the intensity at which participants were exercising (Sajid et al., 2016; Yuen et al., 2019; Zadro et al., 2019). In the study of Sajid et al. (2016), the exergaming group and one comparison group engaged in exercises of similar low to moderate intensity. In contrast, participants in the study of Yuen et al. (2019) played exergames at a moderate to heavy intensity, whereas the control group played a cognitive digital game that was not physically taxing. Participants in the study of Zadro et al. (2019) played exergames at a moderate intensity or continued usual activities (control group). One study (5%) explicitly noted that intensity was at own convenience (Villumsen et al., 2019), and the other 16 (80%) studies did not provide information on exercise intensity.

Third, session duration varied between 15 and 60 min in the exergaming and control/comparison groups. Four studies (20%) reported information on session duration only in terms of minimum or maximum values, three of which only reported prescribed duration per week (session duration unknown). No information on session duration was provided for the exergaming group in overall five studies (25%), and six studies (30%) used control groups that were not instructed to exercise, so session duration was not applicable (see Table 2).

Fourth, the frequency in the studies ranged from two times per week (Punt et al., 2016; Zahedian-Nasab et al., 2021) up to seven days per week (Prosperini et al., 2013; Adie et al., 2017; Ambrosino et al., 2020). In between, participants also played exergames three times per week (Zondervan et al., 2016; Imam et al., 2017; Golla et al., 2018; Villumsen et al., 2019; Yuen et al., 2019; Zadro et al., 2019; Sanders et al., 2022), three to four times per week (Tefertiller et al., 2019), five times per week (Meldrum et al., 2015; Sajid et al., 2016; Jaarsma et al., 2021b), and six times per week (Yacoby et al., 2019). Participants in one study played at their own convenience (Tao et al., 2022), and there was no information about training frequency in one study (Sanders et al., 2020). Notably, one study used different frequency schedules in terms of two times per week in the exergaming group and nine sessions over six weeks in the comparison group (Punt et al., 2016).

Fifth, density of the training remained unclear in all but four studies (20%) (Prosperini et al., 2013; Imam et al., 2017; Zadro et al., 2019; Sanders et al., 2022). Participants had one rest day (Prosperini et al., 2013; Zadro et al., 2019) or one to two rest days in between exercise days (Imam et al., 2017). Another study mentioned that two sets of repetitions were completed in two sessions per day (Sanders et al., 2022).

Sixth, program duration ranged from three weeks (Zondervan et al., 2016; Sanders et al., 2020, 2022) to 12 months (Thomas et al., 2017). Other studies used training programs with a duration of four weeks (Imam et al., 2017; Tao et al., 2022), five weeks (Yacoby et al., 2019), six weeks (Meldrum et al., 2015; Punt et al., 2016; Sajid et al., 2016; Adie et al., 2017; Golla et al., 2018; Zahedian-Nasab et al., 2021), eight weeks (Zadro et al., 2019; Ambrosino et al., 2020), or 12 weeks (Prosperini et al., 2013; Tefertiller et al., 2019; Villumsen et al., 2019; Yuen et al., 2019; Jaarsma et al., 2021b).

Finally, enjoyment regarding the training was assessed in only three studies (15%) (Meldrum et al., 2015; Thomas et al., 2017; Yacoby et al., 2019). Participants who played balance exergames reported more enjoyment than participants in the control group who performed conventional balance exercises (Meldrum et al., 2015). Thomas et al. (2017) found that most participants enjoyed the exergaming intervention while there was no information on the control group (usual care). In the study of Yacoby et al. (2019), enjoyment of participants was high in the exergaming group, and slightly higher than in the comparison group who reported moderate to high enjoyment. Notably, Prosperini et al. (2013) did not report but consider enjoyment since participants could play their favorite games in the last four weeks. Still, enjoyment was neglected in most studies.

An overview of general training principles considered in the studies can be found in Supplementary Table S3. Eight studies (40%) mentioned variation in the exercise and training variables by means of changing exercises or games, four of which (20%) used no systematic manipulation but provided free choice of games. Ten studies (50%) implemented some specificity in the exergaming groups by means of preselected games (Prosperini et al., 2013; Punt et al., 2016; Sajid et al., 2016; Imam et al., 2017; Golla et al., 2018; Tefertiller et al., 2019; Villumsen et al., 2019; Yacoby et al., 2019; Zadro et al., 2019; Zahedian-Nasab et al., 2021). Other studies offered choice of difficulty or adjusted the exercise variables on an individual level or the setting on the group level. Three studies (15%) provided no information on specificity regarding the exergaming group. In the control/comparison groups, specificity was realized in terms of exercise booklets on the group level and tailored exercises in terms of type, difficulty, and intensity on the individual level. Progression was mentioned in 13 studies (65%) and possible by means of change in game levels, game modes, or additional materials, such as resistance bands or free weights. Related to progression and variation, some forms of periodization and programming were realized. No information was provided regarding overload and reversibility (see Supplementary Table S3).

#### 4.2.4. Effects of exergaming on physical health (RQ1)

An overview of effects of exergaming on physical health can be found in Table 3. Physical health improved more in the intervention groups in relation to the comparison groups in seven studies (35%) (Prosperini et al., 2013; Zondervan et al., 2016; Imam et al., 2017; Villumsen et al., 2019; Zadro et al., 2019; Jaarsma et al., 2021a; Zahedian-Nasab et al., 2021). Exergaming was more effective than playing cognitive digital games (Imam et al., 2017), receiving physical activity advice (Jaarsma et al., 2021a), continuing usual activities (Prosperini et al., 2013; Villumsen et al., 2019; Zadro et al., 2019), usual rehabilitation care (Zahedian-Nasab et al., 2021), and conventional exercises (Zondervan et al., 2016). Five studies (25%) reported similar improvement in the groups when compared to tailored exercises (Adie et al., 2017), usual activities (Ambrosino et al., 2020), or conventional exercises (Meldrum et al., 2015; Punt et al., 2016; Tefertiller et al., 2019). Eight studies (40%) reported no significant changes after exergaming and conventional exercises (Golla et al., 2018; Yacoby et al., 2019; Sanders et al., 2020, 2022), playing cognitive digital games (Yuen et al., 2019; Tao et al., 2022), or usual care (Sajid et al., 2016; Thomas et al., 2017). Notably, Sajid et al. (2016) reported no significant changes after exergaming and usual care, but a significant improvement in the comparison group that engaged in a progressive home-based aerobic walking exercise program and a therapeutic resistance band exercise program.

#### 4.2.5. Effects of exergaming on quality of life (RQ2)

As also shown by Table 3, 15 studies reported indicators of quality of life. Seven studies (47%) found higher improvement in the intervention group compared to usual activities (Prosperini et al., 2013; Zadro et al., 2019; Ambrosino et al., 2020), conventional exercises (Punt et al., 2016; Golla et al., 2018), usual rehabilitation care (Zahedian-Nasab et al., 2021), and playing cognitive digital games (Tao et al., 2022). Two studies (13%) reported similar improvement in both groups when compared to tailored exercises (Adie et al., 2017) or conventional exercises (Meldrum et al., 2015). Finally, six studies (40%) reported no significant changes after exergaming and playing cognitive digital games (Imam et al., 2017; Yuen et al., 2019), receiving physical activity advice (Jaarsma et al., 2021a), conventional exercises (Tefertiller et al., 2019), usual care (Thomas et al., 2017), or usual activities (Villumsen et al., 2019).

#### 4.2.6. Experiences with the exergaming interventions (RQ3)

Participant support (RQ3a) was realized in each study and included setting up the exergaming system, training with the exergaming system, and contact with participants (see Table 3). First, the exergaming system was set up by the research team in 10 studies (50%) (Prosperini et al., 2013; Punt et al., 2016; Adie et al., 2017; Golla et al., 2018; Tefertiller et al., 2019; Yacoby et al., 2019; Yuen et al., 2019; Zadro et al., 2019; Jaarsma et al., 2021a; Zahedian-Nasab et al., 2021). The other studies did not provide information on the setup. Hence, although commercial exergames are ready to use, care was taken of the correct setup in these rehabilitation settings. Second, participants received instructions in 10 studies (50%) (Meldrum et al., 2015; Punt et al., 2016; Sajid et al., 2016; Adie et al., 2017; Thomas et al., 2017; Golla et al., 2018; Villumsen et al., 2019; Zadro et al., 2019; Jaarsma et al., 2021a; Zahedian-Nasab et al., 2021). Instructions included written or personal instructions on the exercises and exergames and how to play them. Third, participants received some training before the start of the exergaming intervention in 16 studies (80%) (Prosperini et al., 2013; Meldrum et al., 2015; Punt et al., 2016; Sajid et al., 2016; Zondervan et al., 2016; Adie et al., 2017; Imam et al., 2017; Thomas et al., 2017; Golla et al., 2018; Tefertiller et al., 2019; Yacoby et al., 2019; Ambrosino et al., 2020; Sanders et al., 2020, 2022; Jaarsma et al., 2021a; Tao et al., 2022). Noteworthy, exergaming started in clinical settings and then transitioned to exergaming in home environments in five studies (25%) (Meldrum et al., 2015; Imam et al., 2017; Thomas et al., 2017; Ambrosino et al., 2020; Tao et al., 2022). Fourth, during the exergaming intervention, participants were contacted via phone in 11 studies (55%) (Sajid et al., 2016; Zondervan et al., 2016; Adie et al., 2017; Imam et al., 2017; Thomas et al., 2017; Golla et al., 2018; Villumsen et al., 2019; Yacoby et al., 2019; Yuen et al., 2019; Zadro et al., 2019; Jaarsma et al., 2021a). In one of these studies, participants had contact in multiple ways (face-to-face, phone, and email) (Thomas et al., 2017). In one study (5%), participants themselves could call the study coordinator in case of technical or health issues (Ambrosino et al., 2020). Moreover, participants had physiotherapist meetings in two studies (10%) (Prosperini et al., 2013; Meldrum et al., 2015). Further, participants in one study (5%) had several contact possibilities since they were living in a nursing home (Zahedian-Nasab et al., 2021). In five studies (25%), participants were not contacted by the research team during the unsupervised phase (Punt et al., 2016; Tefertiller et al., 2019; Sanders et al., 2020, 2022; Tao et al., 2022). Hence, participant support was common but also implemented differently in studies on unsupervised exergaming in home environments.

Adherence with the intervention (RQ3b) was reported in 15 studies (see Table 3). Adherence with the intervention was high (>70% of the intended goal) in eight studies (53%) (Prosperini et al., 2013; Meldrum et al., 2015; Adie et al., 2017; Imam et al., 2017; Golla et al., 2018; Villumsen et al., 2019; Zadro et al., 2019; Ambrosino et al., 2020). Adherence was found to be moderate in six studies (40%) (Tefertiller et al., 2019; Yacoby et al., 2019; Sanders et al., 2020, 2022; Jaarsma et al., 2021a; Tao et al., 2022) and low (<30%) in one study (7%) (Yuen et al., 2019). In two studies, adherence was not assessable since participants did not receive a specific goal, such as the frequency or duration of exergaming (Thomas et al., 2017), or since exact adherence metrics remained unknown (Zondervan et al., 2016). In sum, adherence to the unsupervised exergaming interventions was moderate to high in most studies.

Information on adverse outcomes (RQ3c) was provided in 14 studies (see Table 3). Adverse outcomes were found in four studies (29%) and included low back pain (Meldrum et al., 2015), mild to moderate low back pain or knee pain (Prosperini et al., 2013), leg and back pain or other non-serious outcomes (Thomas et al., 2017), and non-heart-related chest pain due to surgical clips in the thorax (Villumsen et al., 2019). By contrast, 10 studies (71%) found no adverse outcomes related to exergaming (Adie et al., 2017; Imam et al., 2017; Golla et al., 2018; Tefertiller et al., 2019; Yacoby et al., 2019; Yuen et al., 2019; Zadro et al., 2019; Ambrosino et al., 2020; Jaarsma et al., 2021a; Tao et al., 2022). Relatedly, it was found that exergaming resulted in low to moderate stress levels (Golla et al., 2018), satisfaction with the intervention (Punt et al., 2016; Golla et al., 2018; Yacoby et al., 2019), and higher enjoyment compared to conventional exercises (Meldrum et al., 2015). Overall, few studies have found mild to moderate adverse outcomes of unsupervised exergaming.

#### 4.2.7. Financial support and financial conflicts of interest

In the context of commercial exergames, financial support of the studies and financial conflicts of interest of the study authors could be of relevance. Concerning financial support, one study (5%) indicated no funding yet received three exergaming devices from the manufacturer (Zadro et al., 2019), and two studies (10%) received no funding (Prosperini et al., 2013; Ambrosino et al., 2020). The remaining 17 studies (85%) received public funding from universities or other institutions (Meldrum et al., 2015; Punt et al., 2016; Sajid et al., 2016; Zondervan et al., 2016; Adie et al., 2017; Imam et al., 2017; Thomas et al., 2017; Golla et al., 2018; Tefertiller et al., 2019; Villumsen et al., 2019; Yacoby et al., 2019; Yuen et al., 2019; Sanders et al., 2020, 2022; Jaarsma et al., 2021a; Zahedian-Nasab et al., 2021; Tao et al., 2022). A financial conflict of interest was declared by authors of two studies (10%) regarding the manufacturers of the exergaming device (Zondervan et al., 2016; Sanders et al., 2022), and authors of one study (5%) declared a conflict of interest concerning healthcare and pharmaceutical companies (Prosperini et al., 2013). Three studies (15%) contained no information on competing interests (Punt et al., 2016; Yacoby et al., 2019; Yuen et al., 2019), and the remaining 14 studies (70%) declared no conflict of interest (Meldrum et al., 2015; Sajid et al., 2016; Adie et al., 2017; Imam et al., 2017; Thomas et al., 2017; Golla et al., 2018; Tefertiller et al., 2019; Villumsen et al., 2019; Zadro et al., 2019; Ambrosino et al., 2020; Sanders et al., 2020; Jaarsma et al., 2021a; Zahedian-Nasab et al., 2021; Tao et al., 2022). Overall, most studies received financial support in terms of public funding and most authors reported no competing interests.

### 4.3. Quality assessment of included studies

**Figure 2:**
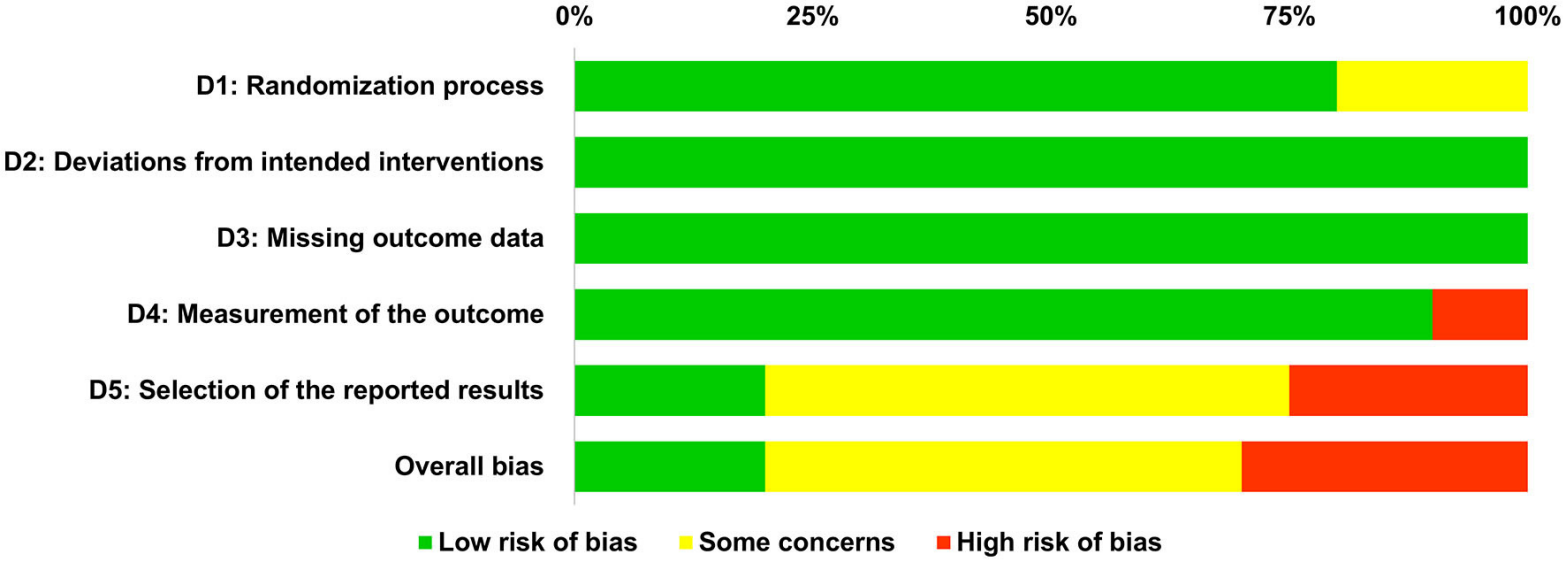
Overview of risk of bias regarding the randomization process, deviations from intended interventions, missing outcome data, measurement of the outcome, selection of the reported results, and overall risk of bias.

The quality assessment of the included studies was based on the revised tool to assess the risk of bias in randomized trials (RoB 2) (Sterne et al., 2019). An overview of the quality assessment for each of the five domains of the risk of bias tool is provided in Figure 2. As shown, the overall risk of bias was rated as low in four studies (20%) (Meldrum et al., 2015; Adie et al., 2017; Villumsen et al., 2019; Sanders et al., 2022), and as high in six studies (30%) (Imam et al., 2017; Golla et al., 2018; Yacoby et al., 2019; Zadro et al., 2019; Sanders et al., 2020; Jaarsma et al., 2021a). Reasons for high risk of bias covered ceiling effects for the primary outcome (Golla et al., 2018) and reporting bias in terms of omitting measures that were announced in the protocol related to the primary outcome (Imam et al., 2017; Yacoby et al., 2019; Zadro et al., 2019; Sanders et al., 2020; Jaarsma et al., 2021a). In addition, some concerns were found in 10 studies (50%) (Prosperini et al., 2013; Punt et al., 2016; Sajid et al., 2016; Zondervan et al., 2016; Thomas et al., 2017; Tefertiller et al., 2019; Yuen et al., 2019; Ambrosino et al., 2020; Zahedian-Nasab et al., 2021; Tao et al., 2022). Reasons for some concerns were a missing protocol (Prosperini et al., 2013; Punt et al., 2016; Tefertiller et al., 2019; Ambrosino et al., 2020), a missing analysis plan in the trial registration (Thomas et al., 2017; Yuen et al., 2019; Zahedian-Nasab et al., 2021), missing information on the randomization process (Sajid et al., 2016; Tefertiller et al., 2019), and reporting bias regarding a secondary outcome (Zondervan et al., 2016; Tao et al., 2022).

**Figure 3:**
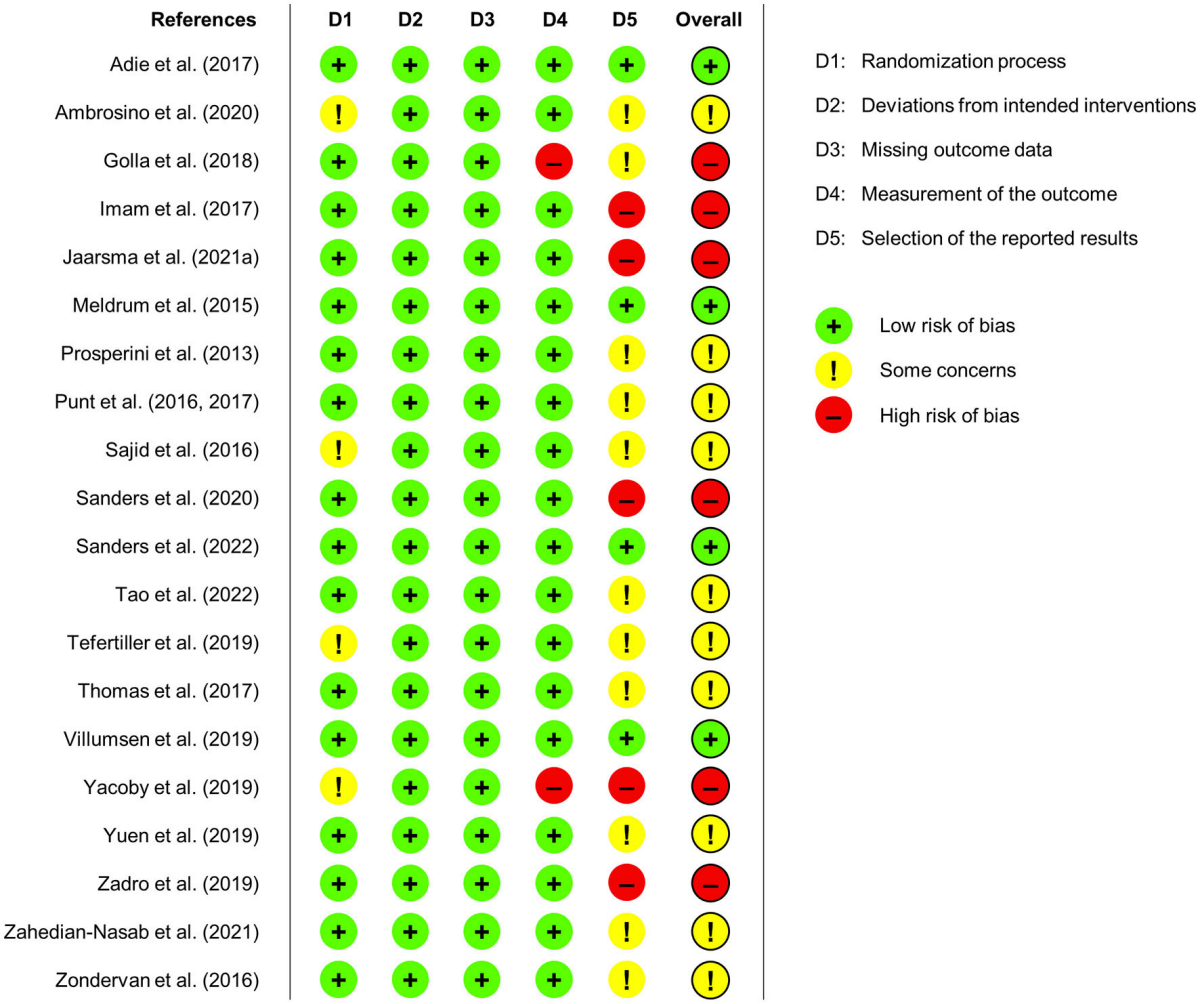
Risk of bias ratings for each of the included studies regarding the randomization process, deviations from intended interventions, missing outcome data, measurement of the outcome, selection of the reported results, and overall risk of bias.

In addition to the overall frequency analysis presented in Figure 2, the results of the quality assessment for each included study are shown in Figure 3. In the following, we explain our ratings for each of the five domains of the risk of bias tool (Sterne et al., 2019): The first domain (D1) considers potential biases arising from the randomization process, which was low for all but four studies that were related to some concerns (Sajid et al., 2016; Tefertiller et al., 2019; Yacoby et al., 2019; Ambrosino et al., 2020). These concerns arose due to the use of block randomization with a fixed block size which can result in a predictable allocation process (Yacoby et al., 2019; Ambrosino et al., 2020), and due to missing exact information on the randomization process (Sajid et al., 2016; Tefertiller et al., 2019). The second domain (D2) is about biases due to deviations from intended interventions, and the third domain (D3) is about bias due to missing outcome data. Risk of bias regarding both domains was rated as low for all studies (see Figures 2, 3). The fourth domain (D4) addresses biases in the measurement of the outcome, which was rated as low for all but two studies that found ceiling effects in three outcome measures at baseline (Golla et al., 2018) or used retrospective self-ratings instead of validated scales to assess improvements in physical health (Yacoby et al., 2019), both resulting in a high risk of bias. The fifth domain (D5) is about risk of bias because of the selection of the reported results, which was rated as high in five studies (Imam et al., 2017; Yacoby et al., 2019; Zadro et al., 2019; Sanders et al., 2020; Jaarsma et al., 2021a). Four studies did not report primary outcomes in the final article that were announced in the trial protocol (Imam et al., 2017; Yacoby et al., 2019; Sanders et al., 2020; Jaarsma et al., 2021a). Zadro et al. (2019) omitted measures that were announced in the protocol and analyzed the data not as specified in the protocol. Moreover, some concerns regarding selection of the reported results were found in 10 studies: Two studies showed some reporting bias in terms of not reporting intended secondary outcomes based on the trial registration (Zondervan et al., 2016; Tao et al., 2022), and reporting measures that were missing in the trial registration (Zondervan et al., 2016). However, as this bias only affected secondary measures, the rating resulted in some concerns instead of high risk of bias. Protocols were missing for five studies (Prosperini et al., 2013; Punt et al., 2016; Sajid et al., 2016; Tefertiller et al., 2019; Ambrosino et al., 2020), and analysis plans were not predetermined in three studies (Thomas et al., 2017; Yuen et al., 2019; Zahedian-Nasab et al., 2021). More specifically, Thomas et al. (2017) stated in their protocol that they tested out data analysis procedures, but the article contains only descriptive statistics as suggested for pilot studies, yielding some concerns instead of high risk of bias. Notably, Meldrum et al. (2015) used linear regression instead of the intended analysis of variance, which yet is basically the same analysis; Sanders et al. (2022) were missing an analysis plan, yet the main results are only descriptive. So, the risk of bias was rated as low for both studies.

Taken together, following the risk of bias approach, we could identify several methodological aspects regarding the robustness of results of the included studies. Nevertheless, we found a low risk of bias for most aspects and most concerns are due to potentially unreported results. Thus, the reported results of the studies included in this review can be considered largely robust.

## 5. Discussion

The delivery of rehabilitation measures will continue to play a key role in meeting the high global demand for rehabilitation services, which requires an increasing number of rehabilitation professionals (cf. Cieza et al., 2020). In this context, the unsupervised use of commercial exergames at home could be a complementary and effective rehabilitation approach to alleviate the lack of professional workforce and reduce healthcare costs. To this end, we provided a systematic review of the available evidence on the effects of the unsupervised use of commercial exergames at home on physical health and quality of life and we will discuss its potential for home rehabilitation in the following.

### 5.1. Effects of unsupervised exergaming at home on physical health (RQ1) and quality of life (RQ2)

Regarding physical health (RQ1), seven of the 20 included studies (35%) found that exergaming was more effective than usual rehabilitation care, usual activities, conventional exercises, cognitive digital games, and physical activity advice. Still, three of these studies were related to a high risk of bias in terms of outcome reporting bias regarding the primary outcome. Moreover, four studies yielded some concerns due to missing protocol and analysis plans so that only one study was related to an overall low risk of bias. In addition, similar improvement was found in five studies (25%) that compared exergaming to tailored exercises, usual activities, or conventional exercises. Three of these studies were related to some concerns, and two were related to low risk of bias. Eight studies (40%) found no significant changes in both groups and ranged from low to high risk of bias. In sum, considering that effects on some primary outcomes remain unclear due to reporting bias, most studies reported similar or higher effects of unsupervised exergaming at home on physical health in relation to comparison interventions.

Concerning the 15 studies that evaluated the effects of exergaming on quality of life (RQ2), seven studies (47%) reported better outcomes for the exergaming groups compared to usual rehabilitation care, usual activities, conventional exercises, and cognitive digital games. Two of these studies had a high risk of bias in terms of ceiling effects or outcome reporting bias regarding the primary outcome. Quality of life was also found to increase similarly in two studies (13%) that compared exergaming to tailored or conventional exercises and had a low risk of bias. No significant changes were found in six studies (40%), which ranged from low to high risk of bias. Overall, most studies reported that the unsupervised use of commercial exergames in home environments had similar or higher beneficial effects on adults' quality of life in relation to comparison interventions.

### 5.2. Experiences with unsupervised exergaming at home (RQ3)

Concerning experiences with the exergaming interventions, we focused on participant support in relation to the intervention (RQ3a), adherence with the intervention (RQ3b), and adverse outcomes related to the intervention (RQ3c).

Participant support was realized in all studies and included setting up the exergaming system, training with the exergaming system, and contact with participants during the unsupervised phase of the intervention. Participant support regarding setting up the exergaming system was reported in 10 studies (50%), whereas the installation remains unknown in the other studies. Additional instructions were mentioned in 10 studies (50%), and training with the exergaming system was reported in 16 studies (80%). Contact with participants during the unsupervised phase of the intervention was most often realized via phone calls from professionals in 11 studies (55%), one of which also included contact via face-to-face and email. In two studies (10%), participants had regular physiotherapist meetings. In one study each (5%), participants were free to contact in case of technical or health issues, or were living in a nursing home. In the remaining five studies (25%), no contact with participants was mentioned regarding the unsupervised phase of the intervention. Overall, each study on unsupervised exergaming at home included some participant support mechanisms to ensure the fidelity of the intervention.

Adherence was reported in 15 studies and was high (>70% of the intended goal) in eight studies (53%), moderate in six studies (40%), and low (<30% of the intended goal) in one study (7%). Twelve of these 15 studies (80%) have made use of diaries and daily play logs to assess adherence, similar to previous research (e.g., Donoso Brown et al., 2020). Several studies included home visits or telephone support to check and ensure adherence, which are also strategies to increase adherence (Simek et al., 2012). In addition, reasons for adherence were examined in more detail for one study with moderate adherence (Jaarsma et al., 2021a). In this study, more adherent participants could motivate themselves to exercise alone, had fewer sleeping problems, and had a higher exercise capacity (Jaarsma et al., 2021b). Moreover, the effects of supervision on adherence seem to remain context-specific. For instance, the secondary analysis of one included study (Imam et al., 2017) emphasizes that supervision could increase the exergaming frequency and duration of adults who have had a lower limb amputation (Tao et al., 2020). However, adults who have had a stroke and engaged in self-directed exergaming were found to exercise twice as long and perform eight times more repetitions compared to standard care (Broderick et al., 2021). Overall, considering that effects of supervision remain context-specific, adherence to exergaming can be high even in unsupervised home environments.

Ten of the 14 studies (71%) that investigated adverse outcomes found no adverse outcomes related to exergaming. The remaining four studies (29%) reported mostly mild and some moderate adverse outcomes, such as back pain or knee pain. These findings are in line with previous work that reported mild to moderate adverse events in few studies on using exergames in supervised and home environments (Prosperini et al., 2021). Moreover, although hand lacerations are a common injury that has been associated with the use of Wii consoles (Sparks et al., 2009), such injuries were not reported by the three included studies that have used it in the context of home rehabilitation (Adie et al., 2017; Thomas et al., 2017; Jaarsma et al., 2021a). Thus, the unsupervised use of commercial exergames at home seems to be mostly safe when some precautions are considered (cf. Threapleton et al., 2016).

### 5.3. Exergaming at home for adults with different rehabilitation needs

In the following, we take a closer look at the pathologies investigated in the studies to discuss for which rehabilitation needs exergaming may be an effective approach. Noteworthy, four pathologies were addressed in multiple studies, i.e., stroke, multiple sclerosis, prostate cancer, and lower limb amputation. Overall, the findings from the included studies suggest that the unsupervised use of commercial exergames in home environments can support the rehabilitation of the physical health and quality of life in adults with these pathologies.

First, adults who have had a stroke and played Wii Sports games were found to improve their physical health in terms of arm function like participants who engaged in tailored arm exercises (Adie et al., 2017). Playing a game that comes with the commercially available device MusicGlove improved gripping function and motor activity even more than conventional hand therapy exercises (Zondervan et al., 2016). However, three studies reported non-significant findings regarding balance and gait after using Wii Fit games (Golla et al., 2018), gripping function after using MusicGlove (Sanders et al., 2020), and upper extremity function and balance based on self-rated improvements after playing Xbox Kinect games or PlayStation EyeToy games (Yacoby et al., 2019). Additionally, playing Wii Sports games and completing tailored arm exercises resulted in a similar improvement in quality of life in terms of health state (Adie et al., 2017), and playing Wii Fit games in higher balance confidence than conventional balance exercises (Golla et al., 2018). Overall, our findings complement meta-analytic results on the effectiveness of exergames for people who have had a stroke (e.g., Unibaso-Markaida and Iraurgi, 2021; Chen et al., 2022; Gelineau et al., 2022; Truijen et al., 2022).

Second, adults with multiple sclerosis were found to significantly improve their physical health in terms of balance and gait measures (Prosperini et al., 2013; Thomas et al., 2017) as well as physical activity (Thomas et al., 2017) after playing Wii Sports games or Wii Fit games regularly for three or more months compared to usual activities or usual care. In these studies, quality of life was also found to improve in terms of less physical and psychological impact of multiple sclerosis (Prosperini et al., 2013; Thomas et al., 2017) as well as more self-efficacy, less self-reported depression, and less hospital anxiety (Thomas et al., 2017). In line with these findings, meta-analytic results highlight the positive effects of exergaming on multiple sclerosis (e.g., Calafiore et al., 2021; Truijen et al., 2022).

Third, one study found that adults with prostate cancer improved their physical health in terms of walking capacity after playing Xbox 360 Kinect games compared to usual activities for 12 weeks (Villumsen et al., 2019). Another study found no significant changes in the exergaming group that played Wii Fit games for six weeks, but a significant increase in physical performance and walking capacity in the comparison group that engaged in progressive exercising for six weeks (Sajid et al., 2016). Additionally, quality of life was numerically higher in terms of global health status (Villumsen et al., 2019), yet neither study provides evidence on significant changes in quality of life.

Fourth, adults who have had a lower limb amputation and played Wii Fit games regularly for four weeks significantly improved their physical health in terms of walking capacity and physical activity, while the validity of the quality of life measure was limited in terms of ceiling effects (Imam et al., 2017). By contrast, adults who have had a lower limb amputation and played Wii Fit games for overall eight weeks showed higher quality of life in terms of balance confidence compared to playing digital cognitive games, but no significant changes in physical health were found (Tao et al., 2022). Notably, the latter two studies refer to the same protocol, yet the study by Tao et al. (2022) started with a supervised phase of four weeks, followed by an unsupervised phase of four weeks during which participants could see other participants playing via tablet devices.

In the other nine studies, one pathology each was investigated, including rheumatoid arthritis, heart failure, unilateral peripheral vestibular loss, ankle sprain, spinal cord injury, traumatic brain injury, idiopathic pulmonary fibrosis, chronic low back pain, and fall risk. First, adults with rheumatoid arthritis played Wii Fit games for eight weeks, while home training after in-hospital training further improved their physical health in terms of global health and quality of life in terms of reduced difficulty with activities (Ambrosino et al., 2020). Second, a regular use of Wii Sports games for 12 weeks was found to increase physical health in terms of muscle function in adults with heart failure, yet no significant changes were found in other measures of physical health and quality of life (Jaarsma et al., 2021a). Third, after having played Wii Fit games for six weeks regularly, adults with unilateral peripheral vestibular loss had better physical health in terms of gait speed and standing balance, and higher quality of life in terms of higher balance confidence as well as lower anxiety and depression (Meldrum et al., 2015). Similar effects were found when participants engaged in conventional balance exercises. Fourth, playing Wii Fit games regularly for six weeks was also of similar or higher effectiveness compared to conventional physical therapy or exercise therapy for adults with an ankle sprain (Punt et al., 2016). In particular, participants showed similar or higher improvements in physical health in terms of foot and ankle ability and temporal-spatial gait parameters as well as similar or higher improvements in quality of life in terms of pain during walking and rest. Fifth, another study reported better physical health in terms of a numerically higher gripping function in adults with spinal cord injury after three weeks of regularly playing a game that comes with the commercially available device MusicGlove compared to conventional hand therapy exercises (Sanders et al., 2022). Sixth, playing Xbox Kinect games regularly for three months could increase physical health in terms of balance more than a traditional home-based exercise program in adults with traumatic brain injury, yet there were no significant changes in quality of life in terms of balance confidence and community participation (Tefertiller et al., 2019). Seventh, adults with idiopathic pulmonary fibrosis did not show a significant increase in physical health or quality of life after playing Wii Fit games at a moderate to high intensity and engaging in physical activity compared to engaging in physical activity for 12 weeks (Yuen et al., 2019). These findings may be due in part to the observed low adherence to the intervention, insufficient patient support, or the games, but these were not specified. Eighth, after playing Wii Fit U games regularly for eight weeks, adults with chronic low back pain showed better physical health in terms of higher physical function and engagement in physical activity (Zadro et al., 2019). Regarding quality of life, pain self-efficacy was higher and pain intensity over the last week was lower in the exergaming group compared to the group that continued usual activities. Finally, adults with fall risk were found to have better physical health in terms of balance and higher quality of life in terms of lower fear of falling after they had played Xbox Kinect sports games regularly for 6 weeks (Zahedian-Nasab et al., 2021). Taken together, these findings are promising and show that the unsupervised use of exergames in home environments can support the rehabilitation of physical health and quality of life in adults with various pathologies.

### 5.4. The role of exercise prescriptions in exergaming at home

The exercise and training variables that we have considered allow discussing possible implications for exercise prescription and the different effects of exergaming on physical health in the included studies.

Regarding the exercise and training variables in the seven studies with more positive effects of exergaming on physical health, participants engaged in moderate intensity exercising in one study (Zadro et al., 2019) and in exercising at own convenience in another study (Villumsen et al., 2019). Participants in the control groups of both studies continued usual activities, while it remains unknown whether these activities included exercising. Information on intensity was missing in the other studies (Prosperini et al., 2013; Zondervan et al., 2016; Imam et al., 2017; Jaarsma et al., 2021a; Zahedian-Nasab et al., 2021). Session durations ranged between 30 min (Prosperini et al., 2013; Jaarsma et al., 2021a), 30–60 min (Zahedian-Nasab et al., 2021), 40 min (Imam et al., 2017), and 60 min (Villumsen et al., 2019; Zadro et al., 2019), whereas one study provided no exact information (Zondervan et al., 2016). The latter study was the only in which participants in the comparison group also engaged in exercising, and the minimum session duration between groups was similar (Zondervan et al., 2016). Exercise frequency ranged from two times to seven days per week, and program duration ranged from three to 12 weeks. Taken together, in most studies with more positive effects of exergaming, information on intensity was missing and session duration, exercise frequency, and program duration varied and cannot be compared between exergaming and control/comparison groups due to the study design.

Regarding the exercise and training variables in the five studies with similar improvements, four studies provided no information on intensity (Meldrum et al., 2015; Adie et al., 2017; Tefertiller et al., 2019; Ambrosino et al., 2020). Punt et al. (2016) implemented a preferred difficulty level in the exergaming group and adjusted the difficulty level to participants' progress in the comparison group. Session durations were similar between groups in three studies and ranged from 15 min (Meldrum et al., 2015), over 30 min (Punt et al., 2016; Tefertiller et al., 2019), to at most 45 min (Adie et al., 2017). In one study with usual activities as the control condition, participants completed a session of 50 min (10 min per game) in the exergaming group (Ambrosino et al., 2020). Exercise frequency ranged from two times to seven days per week, while program duration was most frequently six weeks and ranged up to 12 weeks. Enjoyment was assessed in one study and higher in the exergaming group compared to conventional exercises (Meldrum et al., 2015). In sum, in case of most studies with similar improvements, exercise intensity remains unclear while session duration, exercise frequency, and program duration were similar between exergaming and control/comparison groups that also completed exercises.

Regarding the eight studies with non-significant findings, five studies provided no information on intensity (Thomas et al., 2017; Golla et al., 2018; Sanders et al., 2020, 2022; Tao et al., 2022) and one study specified some information about intensity only in the control group (Yacoby et al., 2019). Sajid et al. (2016) reported that participants exercised at a low to moderate intensity in both groups. Another study stated that participants engaged in moderate to heavy exercising in the exergaming groups compared to digital gaming that was not physically taxing in the control group (Yuen et al., 2019). Session duration remained unknown in terms of at least 3 h a week (Sanders et al., 2020, 2022), as much as participants liked (Tao et al., 2022), or no information on session duration (Sajid et al., 2016; Thomas et al., 2017). In the remaining three studies, session durations were similar between groups and ranged from 30 min (Golla et al., 2018) to 60 min in both groups (Yacoby et al., 2019), also by means of combining 30 min of play and 30 min of additional physical activity (Yuen et al., 2019). Exercise frequency ranged from three to six times per week, and program duration ranged from three to 12 months. Enjoyment was assessed in two studies and most participants enjoyed the exergaming intervention in one study (Thomas et al., 2017), while enjoyment was slightly higher in the exergaming group compared to a conventional self-training program in another study (Yacoby et al., 2019). So, studies with non-significant findings included few information on intensity and session duration as well as different exercise frequencies and program durations, which complicates to understand the missing effectiveness in both groups.

Taken together, the included studies lack information regarding several exercise and training variables, which complicates to formulate exercise prescriptions for home-based exergaming. Still, studies that missed information on exercise prescriptions found positive effects on physical health and quality of life. It could therefore be that in these studies participants exercised at their own convenience, so that unsupervised home-based exergaming might be beneficial for adults' physical health and quality of life even in the absence of detailed exercise prescriptions. Overall, several exercise prescriptions may yield positive effects of exergaming in people with different pathologies.

### 5.5. Implications for research and practice

This systematic review of randomized controlled trials shows that playing commercial exergames in home environments can effectively support rehabilitation measures toward physical health and quality of life. Our findings extend evidence from previous systematic reviews regarding these outcomes and provide valuable information about the characteristics of the home-based interventions.

With regard to physical health as the primary target outcome of physical rehabilitation, other systematic reviews found that the use of commercial exergames can have beneficial effects compared to conventional care and other groups on balance in adults with neurological pathologies (Prosperini et al., 2021; Unibaso-Markaida and Iraurgi, 2021) and on several physical health outcomes in older adults above the age of 65 living in long-term care homes (Chu et al., 2021). Potential improvements in motor functions have been reported in a previous systematic review on the use of commercial exergames in several physical rehabilitation settings; yet data have been lacking on potential adverse effects of exergaming (Bonnechère et al., 2016). Additionally, some of these systematic reviews contain findings based on various study designs in lack of a risk of bias assessment (Bonnechère et al., 2016) or findings related to a high risk of bias in most of the studies (Chu et al., 2021), limiting the possibility to draw practical implications. Other works provided evidence on adults with neurological pathologies who used exergames in different settings (Prosperini et al., 2021; Unibaso-Markaida and Iraurgi, 2021). In this context, our systematic review shows that the unsupervised use of commercial exergames at home can have similar or larger beneficial effects on the physical health of adults with different pathologies. These findings are based on experimental evidence with hardly high risk of bias and few adverse outcomes, indicating that home-based exergaming interventions can be considered effective, feasible, and mostly safe in the context of physical rehabilitation.

Regarding quality of life as a secondary outcome of physical rehabilitation, effects of exergaming on quality of life have been considered before. However, based on previous systematic reviews, quality of life outcomes were only considered in relatively few observational and experimental studies; quality of life was only higher after exergaming in few of these studies compared to conventional rehabilitation in adults who have had a stroke (Unibaso-Markaida and Iraurgi, 2021) and several comparison interventions for older adults above the age of 65 with various pathologies (Cacciata et al., 2019; Chu et al., 2021). A more recent meta-analysis found that the use of commercial exergames improved the health-related quality of life in adults with chronic diseases in home-based settings compared to conventional care (Cugusi et al., 2021). In comparison with these works, our systematic review provides experimental evidence that the unsupervised use of commercial exergames can have similar or larger beneficial effects on the quality of life in adults with different needs for physical rehabilitation.

Concerning the practical question which exergames may yield such beneficial effects, three groups of exergames were used in the studies included in our systematic review. First, 13 and thus most of the included studies (65%) used Wii hardware and software, 10 of which used Wii Fit games (50%). Relatedly, several therapists found the Wii Fit to be a motivating and effective tool to complement conventional therapy, for instance, regarding weight shift and balance training (Imam et al., 2018). Moreover, participants in one study also reported a high usability, exercise variety, and challenge concerning the Wii Fit games (Zadro et al., 2019). Still, perceived barriers include a lack of time and familiarity with games. In sum, playing Wii games resulted in similar or higher effects than comparison groups regarding physical health in all but five studies (Sajid et al., 2016; Thomas et al., 2017; Golla et al., 2018; Yuen et al., 2019; Tao et al., 2022). More specifically, playing Wii games was found to more effectively increase physical health than playing cognitive digital games, receiving physical activity advice, and continuing usual activities (Prosperini et al., 2013; Imam et al., 2017; Zadro et al., 2019; Jaarsma et al., 2021a). Improvements were similar when compared to tailored exercises, usual activities, or conventional exercises (Meldrum et al., 2015; Punt et al., 2016; Adie et al., 2017; Ambrosino et al., 2020). In addition, playing Wii games resulted in similar or higher effects than comparison groups regarding quality of life in all but two studies (Imam et al., 2017; Yuen et al., 2019). In particular, playing Wii games more effectively increased quality of life than usual activities, usual rehabilitation care, conventional exercises, and playing cognitive digital games (Prosperini et al., 2013; Punt et al., 2016; Golla et al., 2018; Ambrosino et al., 2020; Tao et al., 2022). Improvements were similar when compared to tailored exercises or conventional exercises (Meldrum et al., 2015; Adie et al., 2017). Second, Xbox Kinect games only were used in three studies (15%), which reported similar or higher effects regarding physical health compared to usual rehabilitation care, conventional exercises, and usual activities (Tefertiller et al., 2019; Villumsen et al., 2019; Zahedian-Nasab et al., 2021), and regarding quality of life compared to usual rehabilitation care (Zahedian-Nasab et al., 2021). One study (5%) used Xbox games when participants could play while standing or PlayStation EyeToy games when participants could play while sitting and reported non-significant results regarding physical health (Yacoby et al., 2019). Third, a game that comes with the commercially available device MusicGlove was used in the remaining three studies (15%), one of which found higher effects on physical health compared to conventional exercises (Zondervan et al., 2016), while effects on quality of life were not reported.

To turn the potential of using commercial exergames into actual positive effects on physical health and quality of life, some more practical aspects need to be considered. In all of the included studies, participants received support from researchers or therapists to some degree, including the setup of the exergaming system, instructions and training regarding exergaming, and possibilities to contact researchers or therapists. In five studies, exergaming started in a clinical setting and then transitioned to the home setting. Thus, it needs to be considered in practice how (independently) exergaming is initiated and adhered to, which also depends on whether the intervention is affordable. Concerning the largest study included in this systematic review (Jaarsma et al., 2021a), it was shown that the costs of using commercial exergames for rehabilitation were relatively low and that adults with a relatively high salary were willing to pay more than half of the intervention costs (Klompstra et al., 2022). In this regard, the financing and willingness to pay might be different in middle- and low-income countries (cf. WHO, 2021). Still, the costs for using commercial exergames for rehabilitation are much lower compared to center-based rehabilitation and telerehabilitation (Klompstra et al., 2022). Given that the necessary financial resources are available, exergaming at home can already be initiated when people are in health care and rehabilitation facilities, and contribute to monitoring and decision-making of adults' physical health and quality of life during rehabilitation and beyond (cf. Mura et al., 2022). Throughout the intervention, adherence techniques that have proven successful in studies could also be considered and adapted to yield intended effects. Specifically, effective ways to increase adherence include appropriate instructions, adequate difficulty levels, regular exercise schedules, and individualization of the interventions (see, e.g., Donoso Brown et al., 2020; Ramos Muñoz et al., 2022). In addition, adherence could be improved if people receive social support and can choose between several suitable commercial exergames so that they are provided with variety and new experiences (cf. Rüth and Kaspar, 2021). Relatedly, social support could also complement and improve participant support that was provided in most of the studies to some degree. In sum, adequate adherence measures and support mechanisms should be considered concerning the unsupervised use of commercial exergames in home environments.

Depending on the pathology and individual needs, it should also be considered whether there are suitable customized exergames available. For instance, customized exergames were found to be more effective than commercial exergames concerning the quality of life in adults who have had a stroke (e.g., Chen et al., 2022). Moreover, exergaming at home could be tailored to people's needs and behavior to provide them with personalized user experiences (cf. Gómez-Portes et al., 2021). Notably, customization of exergames also includes taking precautions to avoid adverse events by using specialized mats or safety harnesses (Zahedian-Nasab et al., 2021; Subramaniam et al., 2022), adjusting exercise intensity by using free weights (Villumsen et al., 2019), or making use of other supportive equipment. Thus, commercial and customized exergames as well as customization of commercial exergames could be considered in practice. More generally, (more) effective exergames could be designed, for instance, by means of process models that consider the target behavior, motivational aspects, game mechanics, and mode of delivery (Robertson et al., 2021). In this regard, several research questions and future directions for the design of effective exergames have also been outlined (Baranowski et al., 2019; Rüth and Kaspar, 2021). Overall, our findings emphasize the effectiveness and feasibility of unsupervised uses of commercial exergames at home regarding physical health and quality of life, while context-specific characteristics should be considered.

### 5.6. Limitations and future research

This work comes with some limitations. First, this systematic review does not include studies on more recent commercial exergames that will be addressed in ongoing or future studies according to study protocols (e.g., Leonardo et al., 2021). Relatedly, the software and hardware for several of the commercial exergames used in the included studies may still be available, but has been discontinued and replaced by the manufacturer with newer products. Such developments are common in the market so that solutions such as software compatibility are needed to provide users a longer-term access to exergames. Moreover, three of the included studies did not provide information on the exact exergames used (Sajid et al., 2016; Thomas et al., 2017; Yuen et al., 2019). Future research should provide sufficient information on the exergames used to facilitate comparisons between interventions and studies, and to facilitate concrete recommendations. Hence, future studies and reviews will remain necessary to examine the effects of the unsupervised use of existing and future commercial exergames in home environments.

Second, while we highlight the overall importance of rehabilitation measures, this review focused on adults who had a diagnosed pathology and a need for physical rehabilitation. The same pathology (stroke) was investigated in at most four studies, nine pathologies were investigated by one study each, and there are other pathologies to take into account. So, the included studies can still be considered pilot work and indicate the need for further research. Moreover, it should be noted that exergaming was found to have positive effects on healthy adults, indicating the potential of exergaming for prevention of pathologies and maintenance of physical activity (Hai et al., 2022). Moreover, exergames can be used in the overlapping areas of rehabilitation, training, and wellness as shown by another systematic review on the use of exergames for older adults above the age of 50 (Kappen et al., 2019). While our systematic review focused on adults, other systematic reviews have reported beneficial effects of home-based exergaming on physical activity and body composition also in younger people (Gao et al., 2020; Oliveira et al., 2020). Thus, future research could take a holistic view on the use of exergames to support people of all ages from their stay in rehabilitation facilities to the transition home and beyond (cf. Mura et al., 2022).

Third, we focused on studies in home environments that evaluated the effects of unsupervised exergaming, including studies with supervised phases (e.g., exergaming started in a clinic and was continued at home). Hence, our review emphasizes that the integration of unsupervised exergaming phases can be an effective and cost-efficient way compared to conventional rehabilitation and supervised exergaming. Still, supervision and participant support were realized in different ways and contexts, for instance, in a clinic (Meldrum et al., 2015), in a nursing home (Zahedian-Nasab et al., 2021), or by means of telerehabilitation (Tao et al., 2022). In addition, the potential benefits of supervision and social support should not be neglected (e.g., Tao et al., 2020; Rüth and Kaspar, 2021). Hence, future research is needed on the appropriate timing and types of participant support to initiate and facilitate unsupervised exergaming at home.

Fourth, our systematic review provides an overview of the available evidence based on certain research approaches, contexts, and instruments. Depending on the pathology and rehabilitation need, specific health-related and exercise-related outcomes as well as moderation and mediation effects might be of interest for the delivery of an intervention. Regarding experiences with the intervention, the aspect of enjoyment was considered only in three studies (15%), although enjoyment and fun have been discussed as integral parts in exergaming and physical activity to elicit health benefits (Mellecker et al., 2013). Relatedly, future research could make use of available validated instruments, for instance, the motivation for exergame play inventory of Staiano et al. (2019). Overall, few studies reported psychological aspects of exergaming, which could receive stronger consideration in future research.

Finally and more generally, the term exergame or active video game was used in only six of the included studies (Villumsen et al., 2019; Yacoby et al., 2019; Yuen et al., 2019; Ambrosino et al., 2020; Jaarsma et al., 2021a; Zahedian-Nasab et al., 2021). Moreover, several of the included studies used the more general term virtual reality instead of more specific terms, such as exergames or active video games (Prosperini et al., 2013; Meldrum et al., 2015; Punt et al., 2016; Adie et al., 2017; Thomas et al., 2017; Tefertiller et al., 2019). In addition to these known terminological pitfalls that we had to anticipate with our search strategy, most of the included works did not refer to theoretical approaches for why exergames may support rehabilitation. Such a lack of terminological clarity and theoretical references has been a general issue in the field of exergames and digital technology (Rüth and Kaspar, 2017; Benzing and Schmidt, 2018). Hence, future research could benefit from more terminological clarity and consideration of other recommendations for research on exergames and beyond (e.g., Threapleton et al., 2016; Rüth and Kaspar, 2017, 2021; Benzing and Schmidt, 2018).

## 6. Conclusions

Commercial exergames can be valuable tools to address the need for physical rehabilitation of people with several pathologies. This work complements previous pathology-specific systematic reviews by providing an overview of the effects of the unsupervised use of commercial exergames on physical health and quality of life in the context of different pathologies. Most of the studies included in our systematic review reported more positive or similar effects of the unsupervised use of exergames in home environments on adults' physical health and quality of life compared to different comparison conditions, such as conventional exercises and usual activities. Some of these studies were related to a high risk of bias due to outcome reporting bias, yet the risk of bias was low or moderate for most studies. More research is needed to formulate clear recommendations regarding exercise prescriptions and to better understand psychological outcomes such as enjoyment of the intervention. To conclude, this systematic review suggests that the unsupervised use of commercial exergames in home environments can be a promising complementary way to address high rehabilitation needs and specifically to improve the physical health and quality of life in adults with different needs for physical rehabilitation.

## Data availability statement

The original contributions presented in the study are included in the article/Supplementary material, further inquiries can be directed to the corresponding author.

## Author contributions

MR, MS, and KK conceptualized the study idea and protocol, which was part of a thesis of MS under the supervision of MR and KK. MR, MS, and KB performed the literature search and coded the included studies. MR wrote the original draft. MR and KB (lead) and MS and KK (supporting) revised the manuscript. All authors approved submission.
