# Supplementary material for: Commercial exergames for rehabilitation of physical health and quality of life: a systematic review of randomized controlled trials with adults in unsupervised home environments
Source: Front Psychol. 2023 Jun 2;14:1155569. doi: 10.3389/fpsyg.2023.1155569 (PMC10272737; doi:10.3389/fpsyg.2023.1155569)
Supplement: Supplementary file 1 [file Data_Sheet_1.pdf]

Supplementary Materials

We conducted the systematic literature review based on the databases Web of Science Core Collection, PsycINFO (via EBSCO), and PubMed, Embase, and CINAHL (via CENTRAL). We made use of available database filters to identify journal articles and randomized controlled trials (RCTs) in English. The exact search strings were as follows:

Table S1. Database search strategy.

**Web of Science Core Collection:**
ALL=((exergam* OR (exercise AND (game* OR gaming)) OR ("virtual reality" OR "augmented reality" OR "mixed reality" OR “mobile” (game* OR gaming)) OR "active video game" OR "interactive video game" OR exertainment OR "activity promoting video game" OR ("digital exercis*") OR game-based OR Kinect OR Wii) AND rehabilitation AND home NOT children NOT (supervis*) NOT review AND (physical* OR health* OR "physical health" OR quality OR "quality of life"))
**PsycINFO (via EBSCO):**
(exergam* OR (exercise AND (game* OR gaming)) OR ("virtual reality" OR "augmented reality" OR "mixed reality" OR “mobile” AND (game* OR gaming)) OR "active video game" OR "interactive video game" OR exertainment OR "activity promoting video game" OR ("digital exercis*") OR game-based OR Kinect OR Wii) AND rehabilitation AND home NOT children NOT (supervis*) NOT review AND (physical* OR health* OR "physical health" OR quality OR "quality of life")
**Pubmed, Embase, and CINAHL (via CENTRAL):**
(exergam* OR (exercise AND (game* OR gaming)) OR ("virtual reality" OR "augmented reality" OR "mixed reality" OR “mobile” AND (game* OR gaming)) OR "active video game" OR "interactive video game" OR exertainment OR "activity promoting video game" OR (("digital" NEXT exercis*)) OR game-based OR Kinect OR Wii) AND rehabilitation AND home NOT children NOT (supervis*) NOT review AND English AND (physical* OR health* OR "physical health" OR quality OR "quality of life") AND (Article:pt OR "Proceedings Paper":pt OR Book:pt OR "Book Chapter":pt OR "Data Paper":pt OR "Abstract of Published Item":pt)

Table S2. Characteristics of the randomized controlled trials included in this systematic review.

Study	Design	Participants		Exergaming intervention	Control/ Comparison	Outcome definition and measurement	Main findings
Author(s) and location(s)	RCT type (blinding)<br><br>Randomization procedures	Pathology (diagnostic criteria)	Sample characteristics (recruitment, sample size, age, gender)	Study implementation: background and aim, devices/exergames used, intended protocol, participant support (RQ3a)	Realization of the control/ comparison group(s)	Physical health (RQ1), quality of life (RQ2), and experiences with the intervention: adherence (RQ3b) and adverse outcomes (RQ3c)	Physical health (RQ1), quality of life (RQ2), and experiences with the intervention: adherence (RQ3b) and adverse outcomes (RQ3c)
Adie et al. (2017)<br><br>Secondary analysis: Wingham et al. (2015)<br><br>United Kingdom	Parallel multicenter RCT (assessor-blinded)<br><br>Permuted block randomization with varying block size	Arm weaknesses following a stroke (Medical Research Council Scale power less than 5 in any joint plane and able to manipulate the remote control)	235 adults from 10 stroke centers.<br><br>Baseline sample:<br>*n* = 235<br>(IG: 117;<br>CG: 118).<br><br>Mean age (standard deviation):<br>• IG: 66.8 years (14.6)<br>• CG: 68.0 years (11.9)<br><br>Gender:<br>• 104 females<br>• 131 males<br><br>Final/analyzed sample:<br>*n* = 209<br>(IG: 101;<br>CG: 108).	Background and aim:<br>Based on a previous systematic review and meta-analysis, studies with larger sample sizes were needed on the effectiveness of commercial exergames in rehabilitation settings. This exergaming intervention aimed to support home-based rehabilitation for people with affected arm function after stroke.<br><br>Devices/exergames used:<br>Participants used a Wii console and were free to play any of the Wii Sports games (bowling, tennis, golf, baseball).<br><br>Intended protocol:<br>Participants were asked to play independently at home for at most 45 min a day for 6 weeks.<br><br>Participant support:<br>• Setup: the gaming system was installed by a research therapist.<br>• Instructions: participants received written instructions and were asked to perform warm-up exercises for 15 min to minimize the risk of injury.<br>• Training: a research therapist with unknown background visited participants at home and showed them how to use the gaming system. Beyond that, participants carried out the intervention independently.<br>• Contact: participants were encouraged to complete their diary via weekly phone calls by a non-blinded researcher	CG did tailored arm exercises based on the Graded Repetitive Arm Supplementary Program (GRASP).	Physical health:<br>• Arm function (ARAT)<br>• Motor activity log<br>• Occupational performance (COPM)<br>• Stroke impairment (MRS)<br><br>Quality of life:<br>• Health-related quality of life (EQ-5D-3L visual analogue scale)<br>• Stroke impact scale (SIS)<br><br>Experiences:<br>• Diary to record daily arm exercises (adherence) and adverse events.<br><br>Physical health and quality of life were measured at baseline, after 6 weeks, and after 6 months.	Physical health:<br>• Arm function was improved in both groups after 6 weeks and 6 months (no significant between-group differences).<br><br>Quality of life:<br>• Both groups reported better health state (no significant between-group differences).<br><br>Experiences:<br>• Adherence was moderate to good: Average daily exercise time was higher in the IG (M = 37 min, SD = 16.2) compared to the CG (M = 32 min, SD = 11.9) (at most 45 min a day were intended).<br>• Adverse outcomes: none related to exergaming.<br><br>Note: assessor-blinding was not successful.

Ambrosino et al. (2020)<br><br>Italy	Parallel pilot RCT (blinding unknown)<br><br>Block randomization (block size: 4)	Rheumatoid arthritis (1987 American College of Rheumato-logy revised criteria)	40 adults (consecutive inpatients) aged between 18-35 years.<br><br>Baseline and final/analyzed sample:<br>*n* = 40<br>(IG: 20;<br>CG: 20).<br><br>Mean age (standard deviation):<br>• IG: 27.05 years (5.71)<br>• CG: 27.85 years (3.41)<br><br>Gender:<br>• 25 females<br>• 15 males	Background and aim:<br>Evidence was lacking on the effectiveness of commercial exergames in rheumatic patients, and it was assumed that exergames could motivate younger people in particular to keep exercising after in-hospital rehabilitation. This exergaming intervention aimed to support home-based rehabilitation for people with rheumatoid arthritis.<br><br>Devices/exergames used:<br>Participants used a Wii Fit balance board independently at home to play aerobics and balance Wii Fit games (running, skiing, balloons shooting, bike slalom, balls moving through labyrinth). Games were selected in advance to activate more muscle clusters in absolute number.<br><br>Intended protocol:<br>Participants were asked to play individually at home for 50 min (10 min per game) once a day for 8 weeks.<br><br>Participant support:<br>• Training: both groups received progressive in-hospital comprehensive rehabilitation program training (provided by physicians, occupational therapists, and exercise physiologists) including exergaming for 1 month, and continued the training independently for additional 2 months at home.<br>• Contact: participants could call the study coordinator in case of technical issues.	CG continued usual activities.	Physical health:<br>• Disease activity (DAS)<br><br>Quality of life:<br>• Fatigue (FACIT)<br>• Patients' difficulty with 20 specific activities of daily living (HAQ)<br><br>Experiences:<br>• Adherence: participants completed a diary and reported playing times at follow-up.<br>• Adverse outcomes: participants could call the study coordinator.<br><br>Physical health and quality of life were measured at baseline, after 1 month (in-hospital training), and after 2 months (one additional month of home training).	Physical health:<br>• Both groups had better physical health after in-hospital training. Home-based training (IG) further improved global health, while the global health of CG returned to baseline.<br><br>Quality of life:<br>• Both groups reported better quality of life after in-hospital training. Home-based training (IG) further improved quality of life in terms less difficulty with activities, but also reported more fatigue than CG, which returned to baseline measures.<br><br>Experiences:<br>• Adherence was very good: On average, participants played 10 to 15 min per videogame/day (10 min per game, once/daily were intended).<br>• Adverse outcomes: no technical or health issues.

Golla et al. (2018)<br><br>Germany	Parallel open-label pilot RCT (external blinded randomization)<br><br>Random assignment in 1:1 ratio	Stroke (ICD-10: I61 or I63)	Of 14 adults (consecutive inpatients) aged ≥ 60 years, 11 completed the study.<br><br>Baseline sample:<br>n = 14.<br><br>Final/analyzed sample:<br>*n* = 11<br>(IG: 6;<br>CG: 5).<br><br>Mean age (standard deviation):<br>• IG: 74.6 years (10.11)<br>• CG: 73.5 years (7.06)<br><br>Gender:<br>• 4 females<br>• 7 males	Background and aim:<br>Evidence was lacking on the feasibility and effectiveness of using commercial exergames at home in people who have had stroke. This exergaming intervention aimed to support home-based rehabilitation for people who have had stroke.<br><br>Devices/exergames used:<br>Participants used a Wii Fit balance board and were free to play 4 Wii Fit Plus balance games (ski slalom, table tilt, penguin slide, balance bubble).<br><br>Intended protocol:<br>Participants were asked to play independently at home at least three times per week for 30 min for 6 weeks.<br><br>Participant support:<br>• Setup: a sport therapist installed the Wii console at participants' home.<br>• Instructions: a sport therapist instructed participants during the supervised phase at the study center and during the one-time home visit before the start of the unsupervised phase.<br>• Training: the intervention was preceded by a 6-week supervised balance training at the study center that consisted of at most 5 individual 60-min sessions with one session per week.<br>• Contact: the sport therapist contacted participants weekly via phone.	CG did conventional balance exercises based on the Otago Exercise Program (primarily balance, no strength exercises: simple balance exercises while standing and walking, e.g., weight-shifting, hip rotation, tandem/single-leg standing, tandem/back-ward walking, heel raises and heel/toe walking).	Physical health:<br>• Balance (BBS, TUG)<br>• Gait activities (DGI)<br>• Posturographic measures<br><br>Quality of life:<br>• Balance confidence (ABC)<br><br>Experiences:<br>• 10-item questionnaire (experience of stress, satisfaction, perceived effects)<br>• Adherence: study completion; exercise sessions and duration (exercise diary)<br>• Adverse outcomes: (self-reported) intervention-related injuries or falls.<br><br>Physical health and quality of life were measured at baseline, after 6 weeks, and after 3 months. Experiences were measured after 3 months (that is after 6 weeks of home-training).	Physical health:<br>• No significant within-group changes in balance and gait (ceiling effects at baseline).<br><br>Quality of life:<br>• Balance confidence improved in the IG from week 6 to week 12 (ceiling effects at baseline).<br><br>Experiences:<br>• Adherence was good to very good: 11 out of 14 persons completed the study; on average, IG completed 23.4 (SD = 6.2) exercise sessions with a mean total duration of 14.8 h (8.9) in 6 weeks. CG completed 16.2 (3.2) exercise sessions with a mean total duration of 7.8 h (1.5) in 6 weeks (at least 24 times or at least 9 h in 6 weeks were intended).<br>• Adverse outcomes: none<br>• Both groups evaluated the unsupervised training as effective, satisfactory, and feasible for self-application. Both groups perceived low to moderate stress, and moderate to high effects (higher values in CG).

Imam et al. (2017)<br><br>Secondary analysis: Tao et al. (2020)<br><br>Canada	Parallel feasibility RCT (assessor-blinded)<br><br>Block randomization with varying block size in a 1:1 ratio	Lower limb amputation (≥ 1 year post a unilateral transtibial or transfemoral amputation; using a prosthesis for ≥ 2 h per day for the past 6 months)	28 adults (inpatients or former inpatients) aged ≥ 50.<br><br>Baseline and final/analyzed sample:<br>*n* = 28 (IG:14; CG: 14).<br><br>Median age (range):<br>• IG: 61.5 years (50–78)<br>• CG: 62.5 years (50–78)<br><br>Gender:<br>• 6 females<br>• 18 males	Background and aim:<br>The feasibility of a training program that includes commercial exergames for improving walking capacity needed to be assessed. The program is based on social cognitive theory and involves all four sources of self-efficacy. This exergaming intervention aimed to improve walking capacity in older adults with lower limb amputation.<br><br>Devices/exergames used:<br>Participants used a Wii Fit balance board to play selected games, including yoga, balance games, strength training, and aerobics.<br><br>Intended protocol:<br>Participants were asked to play independently at home for 40 min 3 times a week (Mondays, Wednesdays, and Fridays) for 4 weeks. Participants should play advanced levels after they had unlocked them in the games.<br><br>Participant support:<br>• Training: the intervention started in the clinic (participants played in groups of 3) and continued in participants' home (participants played independently). One or two high back chairs were used to minimize fall risk in the clinic.<br>• Contact: a trainer contacted the participants weekly via phone to monitor for safety and equipment function.	CG played cognitive digital games (Wii Big Brain Academy Degree), and solved cognitive tasks (identify, memorize, analyze, compute, and visualize)	Physical health:<br>• Walking capacity (2MWT)<br>• Physical activity (PASE)<br>• Mean number of steps taken per day (SAM)<br>• Cognitive-motor interaction (WWT)<br>• Lower limb functioning (SPPB)<br>• Locomotor activities (LCI-5)<br><br>Quality of life:<br>• Balance confidence (ABC)<br><br>Experiences:<br>• Adherence: question in the exit questionnaire on percentage of sessions completed<br>• Adverse outcomes: question in the exit questionnaire<br>• 9 questions on perceived benefit (6-point Likert scale), written and verbal comments, and self-reported pain and fatigue<br><br>Physical health and quality of life were measured at baseline, after 4 weeks, and after 7 weeks. Experiences were measured throughout the trial and at the end of the trial (after 7 weeks).	Physical health:<br>• Only the IG improved in walking capacity after the treatment and at 3-week retention.<br>• IG showed a higher (non-significant) increase in physical activity and mean number of steps taken per day.<br>• Cognitive-motor interaction was better (non-significant) in the IG than the CG after treatment compared to baseline.<br><br>Quality of life:<br>• Ceiling effects<br><br>Experiences:<br>• Adherence was good: Participants completed 83.4% of sessions (12 sessions in 4 weeks were intended).<br>• Adverse outcomes: none<br>• Participants found the intervention useful in improving their walking and liked to continue using the equipment at home.<br>• Participants preferred the group and supervision elements of training in-clinic but liked the convenience and accessibility of in-home training.

Jaarsma et al. (2021a)<br><br>Secondary analysis: Jaarsma et al. (2021b)<br><br>Sweden, Israel, Italy, the Nether-lands, Germany, United States	Parallel multicenter RCT (assessor-blinded, open-label)<br><br>Block randomization (block size: 8 or 12) in a 1:1 ratio	Heart failure (European Society of Cardiology guidelines)	605 adults from 5 university hospitals, one rehabilitation center, 3 regional hospitals, and one cardiology outpatient practice.<br><br>Baseline sample:<br>*n* = 605<br>(IG: 305;<br>CG: 300).<br><br>Mean age (standard deviation):<br>• IG: 66 years (12)<br>• CG: 67 years (11)<br><br>Gender:<br>• 175 females<br>• 430 males<br><br>Final/analyzed sample:<br>*n* = 464<br>(IG: 234;<br>CG: 230).	Background and aim:<br>The work refers to a conceptual model, according to which playing exergames targets people's motivation, physical activity, and self-efficacy. Thereby, exergaming is thought to improve health behaviors, exercise capacity, and health. Evidence was lacking on the effectiveness of using commercial exergames at home in people with heart failure and on their motivation to use it to stay physically active. This exergaming intervention aimed to support home-based rehabilitation for people with heart failure.<br><br>Devices/exergames used:<br>Participants used a Wii to play Wii Sports games (baseball, bowling, boxing, golf, tennis).<br><br>Intended protocol:<br>Participants were asked to play independently at home for 30 min on 5 days per week for 12 weeks, adapted to their individual physical condition.<br><br>Participant support:<br>• Setup: the gaming system was installed by an instructor.<br>• Instructions: participants were instructed to move the remote in similar ways the sport is played in real life.<br>• Training: participants were introduced to the Wii console in a group-based introduction (2 h).<br>• Contact: participants received phone calls at 2, 4, 8 and, 12 weeks to discuss frequency of playing or resolve issues with gaming.	CG received protocol-based physical activity advice.	Physical health:<br>• Walking capacity (6MWT)<br>• Muscle function<br>• Self-reported physical activity<br><br>Quality of life:<br>• Exercise motivation<br>• Exercise self-efficacy<br><br>Experiences:<br>• Adherence: telephone calls and diary<br>• Adverse outcomes: reported spontaneously or observed by the local research team.<br><br>Physical health was measured at baseline, after 3 months, after 6 months, and after 12 months.	Physical health:<br>• Both groups showed non-significant increases in walking capacity (after correction for baseline and confounders).<br>• IG showed higher muscle function (heel-rise left) after 6 months.<br>• No between-group differences in physical activity (yet a descriptive within-group decrease).<br><br>Quality of life:<br>• No between-group differences in exercise motivation and exercise self-efficacy (yet a descriptive within-group decrease).<br><br>Experiences:<br>• Adherence was moderate: 83% used exergames, 45.6% completed the recommended playing time (30 h in 12 weeks were intended).<br>• Adverse outcomes: no major events related to exergaming.

Meldrum et al. (2015)<br><br>Ireland	Parallel RCT (assessor-blinded)<br><br>Permuted block randomization (block size: 6) in a 1:1 ratio	Unilateral peripheral vestibular loss (bithermal caloric irrigation and a canal paresis > 20%; alternative-ly, positive head thrust test, or head shaking after nystagmus, or direction-fixed spontaneous nystagmus)	71 adults (attending the otolaryngology or neurology outpatient clinics).<br><br>Baseline and final/analyzed sample:<br>*n* = 71<br>(IG: 35;<br>CG: 36).<br><br>Mean age (standard deviation):<br>• IG: 57.83 years (13.6)<br>• CG: 50.47 years (15.53)<br><br>Gender:<br>• 44 females<br>• 27 males	Background and aim:<br>Evidence was needed on the effectiveness of using the Wii Fit Plus compared to conventional vestibular rehabilitation in home environments. Participants were expected to complete a progressive training program consisting of gaze stabilization exercises, balance exercises, and a graded walking program. Exergaming was part of the balance exercises. This exergaming intervention (balance program) aimed to support home-based vestibular rehabilitation for people with unilateral peripheral vestibular loss. In this regard, neurophysiological principles regarding the pathology as well as motor learning principles were considered.<br><br>Devices/exergames used:<br>Participants used a Wii Fit Plus balance board, which was modified from a stable to an unstable surface to play several exergames (yoga, leg exercises, balance games, aerobics, training plus games).<br><br>Intended protocol:<br>Participants were asked to complete 30 sessions independently at home. They should play for 15 min on 5 days per week for 6 weeks.<br><br>Participant support:<br>• Instructions: exercises were explained in weekly exercise booklets.<br>• Training: initial training in all exercises was provided in the clinic during weekly treatment sessions (between 4 and 7 sessions).<br>• Contact: participants had weekly physiotherapist appointments (all therapists had completed postgraduate training in vestibular rehabilitation and had an average of 6 years of experience in the rehabilitation of vestibular disorders).	CG did similar gaze stabilization exercises and the walking program as the IG, but conventional balance exercises using a foam balance mat.	Physical health:<br>• Dynamic visual acuity<br>• Gait speed & other gait parameters (step length, step width, percentage of gait cycle spent in double support) (DGI)<br>• Standing balance (SOT)<br><br>Quality of life:<br>• Balance confidence (ABC)<br>• Anxiety and depression (HADS)<br>• Rehabilitation benefits (VRBQ)<br><br>Experiences:<br>• Diary included in weekly exercise booklets to assess weekly adherence and adverse outcomes.<br>• Patient satisfaction with the intervention: 5 items on enjoyment, motivation, adherence, tiredness, difficulty with exercises (5-point Likert scale)<br><br>Physical health, quality of life, and patient satisfaction were measured at baseline, after 2 months, and after 6 months.	Physical health:<br>• IG and CG improved in gait speed and standing balance after 8 weeks and 6 months (no significant between-group differences).<br><br>Quality of life:<br>• IG and CG showed higher balance confidence and lower anxiety and depression (no significant between-group differences). Rehabilitation benefits were non-significantly higher in IG.<br><br>Experiences:<br>• Adherence was good and similar in both groups: The IG completed 77.1% of the total exercises prescribed for each week, the CG completed 78.5% of sessions (30 sessions in 6 weeks were intended).<br>• Adverse outcomes: one related to exergaming (low back pain).<br>• IG reported significantly more enjoyment, less difficulty with and less fatigue after balance exercises.

Prosperini et al. (2013)<br><br>Italy	Crossover pilot RCT<br>(not blinded, no sample size calculation)<br><br>Randomization in a 1:1 ratio to two counter-balanced arms	Multiple Sclerosis (MS)<br>(McDonald revised criteria)	36 adults between 18 and 50 years who regularly attended an MS center.<br><br>Baseline sample:<br>*n* = 36<br>(IG: 18;<br>CG: 18).<br><br>Mean age (standard deviation):<br>• IG: 35.3 years (8.6)<br>• CG: 37.1 years (8.8)<br><br>Gender:<br>• 25 males<br><br>Final/analyzed sample:<br>*n* = 34<br>(IG: 17;<br>CG: 17).	Background and aim:<br>There was lack of experimental evidence on the effectiveness and safety of a home-based balance training using the Wii balance board. This exergaming intervention aimed to support home-based balance rehabilitation for people with multiple sclerosis.<br><br>Devices/exergames used:<br>Participants used a Wii Fit Plus balance board to play up to 7 balance games alone (zazen, table tilt, ski slalom, penguin slide, tightrope walk, soccer heading, balance bubble). For the first 4 weeks, only 3 games were allowed. Participants could play more games afterwards and their favorite games in the last 4 weeks.<br><br>Intended protocol:<br>Participants were asked to complete 48 sessions independently at home: they should play for 30 min on each weekday for 12 weeks but were allowed to skip one session per week.<br><br>Participant support:<br>• Setup: a trained physiotherapist set up the gaming system.<br>• Training: the trained physiotherapist explained the exercise protocol and supervised the first session.<br>• Contact: participants were reminded by physiotherapists to complete the logbook and were encouraged to perform the training. Physiotherapist appointments every 4 weeks to supervise the correct execution of games and to monitor participants' performance.	CG continued usual activities.	Physical health:<br>• Static standing balance (force platform-based measures)<br>• Dynamic standing balance (FSST)<br>• Walking speed (25-FWT)<br><br>Quality of life:<br>• Physical and psychological impact of multiple sclerosis (MSIS-29)<br><br>Experiences:<br>• Diary to log adherence (daily training activity/duration and type of game) and adverse events (occurrence of accidental falls or other events)<br><br>Physical health and quality of life were measured at baseline, after 3 months, and after 6 months.	Physical health:<br>• IG showed higher improvement in static and dynamic balance and walking speed (only in crossover group).<br><br>Quality of life:<br>• IG showed higher improvement in terms of lower physical and psychological impact of multiple sclerosis.<br><br>Experiences:<br>• Adherence was very good. IG: 91.7%, CG: 90.3% of the expected number of sessions (48 sessions were intended).<br>• Adverse outcomes: 5 related to exergaming (2 moderate, 3 mild; knee pain or low back pain).

Punt et al. (2016)<br><br>Secondary analysis: Punt et al. (2017)<br><br>Switzerland	Three-group parallel RCT (assessor-blinded)<br><br>Block randomization	Ankle sprain (clinical examination by an orthopedic consultant and a radiograph)	90 adults (ankle sprain patients discharged from the emergency department of a university hospital).<br><br>Baseline and final/analyzed sample:<br>*n* = 90<br>(IG: 30;<br>CG1: 30;<br>CG2: 30).<br><br>Mean age (standard deviation):<br>• IG: 34.7 years (10.7)<br>• CG1: 34.7 years (11.3)<br>• CG2: 33.5 years (9.5)<br><br>Gender:<br>• 39 females<br>• 51 males	Background and aim:<br>The use of commercial exergames is considered to improve treatment modalities for people with ankle sprains. In this regard, experimental evidence on the effectiveness of the Wii Fit compared to conventional physical therapy was lacking. This intervention aimed to support home-based rehabilitation for people with ankle sprains.<br><br>Devices/exergames used:<br>Participants used a Wii Fit balance board to play 4 balance games (ski slalom, table tilt, penguin slide, balance bubble). Participants were free to choose the difficulty level of the exergames.<br><br>Intended protocol:<br>Participants were asked to play independently at home for at least 30 min twice a week for 6 weeks.<br><br>Participant support:<br>• Instructions: an independent physical therapist provided detailed instructions for how to perform exergaming safely and independently at home.<br>• Training: the physical therapist practiced 4 exergames with participants.	CG1 received conventional physical therapy. Participants completed 9 sessions of 30min over 6 weeks and were advised to practice at home. Exercise difficulty was adjusted to progress of participants.<br><br>CG2 did no exercise therapy and did not receive any further advice.	Physical health:<br>• Foot and ankle ability (FAAM)<br>• Kinematic parameters (maximum dorsiflexion, maximum plantar flexion)<br>• Temporal-spatial gait parameters (gait speed, cadence, step length, single-support time, symmetry index of the step length and symmetry of the single-support time)<br><br>Quality of life:<br>• Pain<br>• Time to return to sport.<br><br>Experiences:<br>• One item on satisfaction (scale from 0 to 4)<br>• One item on perceived effectiveness (scale from 0 to 4)<br><br>Physical health and quality of life were measured at baseline and after 6 weeks. Experiences were measured after 6 weeks. Physical health was measured again after 6 months.	Physical health:<br>• All groups increased foot and ankle ability, gait speed, cadence, and step length after 6 weeks. Only the IG improved in single-support time.<br><br>Quality of life:<br>• After 6 weeks, pain during rest only significantly decreased in the IG. Pain during walking decreased in all groups.<br><br>Experiences:<br>• Adherence: NI<br>• Adverse outcomes: NI<br>• ≥ 85% were (very) satisfied with their treatment and 82% considered the treatment as (very) effective.

Sajid et al. (2016)<br><br>United States	Three-group parallel pilot RCT (investigators, statistician, and data managers were blinded)<br><br>Simple randomization	Prostate cancer (histologi-cally confirmed)	19 older men (patients or former patients of local university hospitals)<br><br>Baseline and final/analyzed sample:<br>*n* = 19<br>(IG: 8;<br>CG1: 6;<br>CG2: 5).<br><br>Mean age (standard deviation):<br>• IG: 77.5 years (6.7)<br>• CG1: 75.7 years (9.5)<br>• CG2: 71.8 years (5.0)	Background and aim:<br>Androgen deprivation therapy is common in people with prostate cancer, yet it causes lean muscle loss and decreases physical performance and functional abilities. Previous works showed that a home-based aerobic and resistance program is effective for people with some pathologies in clinical settings. This home-based exergaming intervention aimed to support physical performance of older men in androgen deprivation therapy.<br><br>Devices/exergames used:<br>Participants used a Wii Fit balance board to play Wii Fit games. Games are not specified and were selected by an American College of Sports Medicine-trained exercise physiologist. The exercise physiologist tailored the exercises to each subject during the baseline assessment. Exercise programs of increasing intensity were unlocked as the patients demonstrated increase in physical performance.<br><br>Intended protocol:<br>Participants were asked to play independently at home on at least 5 days per week for 6 weeks.<br><br>Participant support:<br>• Instructions: participants received an instruction manual.<br>• Training: participants received a single, 45-minute, instructional session with the exercise physiologist.<br>• Contact: weekly reminders were conducted via phone for the first 6 weeks.	CG1 completed a moderately intense progressive home-based aerobic walking exercise program and a tailored, low-to-moderate intensity progressive therapeutic resistance band exercise program.<br><br>CG2 received usual care.	Physical health:<br>• Physical performance (SPPB)<br>• Steps per day (pedometer and diary)<br>• Lean muscle mass (DEXA)<br>• Skeletal muscle strength (handgrip strength and chest press repetitions)<br><br>Quality of life:<br>• NI<br><br>Experiences:<br>• NI<br><br>Physical health was measured at baseline, after 6 weeks, and after 3 months.	Physical health:<br>• Exergaming (IG) did not result in significant changes. However, exercising (CG1) increased physical performance and steps per day compared to usual care (CG2).<br><br>Quality of life:<br>• NI<br><br>Experiences:<br>• Adherence: NI<br>• Adverse outcomes: NI

Sanders et al. (2020)<br><br>United States	Crossover RCT (assessor-blinded)<br><br>Adaptive randomization	Stroke affecting the hand (1–10 weeks post-stroke, stroke subjects admitted at the UCI Medical Center)	17 adults ≤ 80 years<br><br>Baseline sample:<br>*n* = 17<br>(IG: 7;<br>CG: 10).<br><br>Final/analyzed sample:<br>*n* = 11<br>(IG: 6;<br>CG: 5).<br><br>Gender:<br>NI.	Background and aim:<br>Compliance with exercises for home rehabilitation is poor and could be increased by using wearable movement sensors in the chronic phase of stroke. However, evidence is lacking on the feasibility, effectiveness, and compliance of the MusicGlove tool for people in the subacute phase after stroke. This exergaming intervention aimed to support home-based hand rehabilitation for people in the subacute period after stroke.<br><br>Devices/exergames used:<br>Participants used a MusicGlove device and a tablet computer. Participants were free to determine the difficulty level and game mode.<br><br>Intended protocol:<br>Participants were asked to play independently at home for at least 3 h per week for 3 consecutive weeks (9 h in total).<br><br>Participant support:<br>• Setup: the exergame was preinstalled on a tablet computer<br>• Training: participants received 30 min of training on how to use a MusicGlove and tablet computer. A therapist showed participants how to play the games and how to correctly perform the exercises at home.	CG received conventional hand therapy exercises using a booklet.	Physical health:<br>• Gripping function (BBT)<br><br>Quality of life:<br>• NI<br><br>Experiences:<br>• Adherence: computer log data	Physical health:<br>• Descriptively, IG improved more in gripping function.<br><br>Quality of life:<br>• NI<br><br>Experiences:<br>• Adherence was moderate: Average usage duration was 4.1 h (3.2) (46%) (9 h were intended).<br>• Adverse outcomes: NI

Sanders et al. (2022)<br><br>United States	Crossover RCT (assessor-blinded)<br><br>Adaptive randomization	Spinal cord injury affecting hand function (since ≥ 6 months prior to enrollment)	11 adults ≤ 80 years<br><br>Baseline sample:<br>*n* = 11<br>(IG: 6;<br>CG: 5).<br><br>Final/analyzed sample:<br>*n* = 10<br>(IG: 5;<br>CG: 5).<br><br>Mean age (standard deviation):<br>• IG: 49.4 years (18.1)<br>• CG:53.2 years (14.8)<br><br>Gender:<br>• 2 females<br>• 8 males	Background and aim:<br>The tool MusicGlove was originally developed for people who have had stroke. Evidence was lacking on the feasibility and effectiveness of using MusicGlove for people with spinal cord injury. This exergaming intervention aimed to support home-based rehabilitation of hand function for people with chronic spinal cord injury.<br><br>Devices/exergames used:<br>Participants used a MusicGlove device and a laptop with the software preinstalled. Participants were free to determine the difficulty level and game mode.<br><br>Intended protocol:<br>Participants were asked to play independently at home for at least 3 h per week over at least 3 sessions per week, for 3 consecutive weeks (9 h in total).<br><br>Participant support:<br>• Setup: the exergame was preinstalled on a computer.<br>• Training: a therapist showed participants how to correctly perform the exercises at home.	CG received conventional hand therapy exercises using a booklet containing 18 standard exercises.	Physical health:<br>• Gripping function (BBT)<br>• Sensorimotor hand function in persons with chronic spinal cord (GRASSP)<br><br>Quality of life:<br>• NI<br><br>Experiences:<br>• Adherence: computer log data	Physical health:<br>• Descriptively, IG improved more in gripping function as well as sensorimotor hand function in terms of prehension ability and performance.<br><br>Quality of life:<br>• NI<br><br>Experiences:<br>• Adherence was moderate to good: Average usage duration was 6.1 h (3.5) (68%) (9 h were intended).<br>• Adverse outcomes: NI

Tao et al. (2022)<br><br>Canada	Parallel RCT (assessor-blinded)<br><br>Block randomization with varying block size in a 1:1 ratio	Lower limb amputation (≥ 1 year post a unilateral transtibial or transfemoral amputation; using a prosthesis for ≥ 2 h per day for the past 6 months)	71 adults aged ≥ 50 who regularly use a lower limb prosthesis.<br><br>Baseline and final/analyzed sample:<br>*n* = 71 (IG:38; CG: 33).<br><br>Mean age (standard deviation):<br>• IG: 66.6 years (7.5)<br>• CG: 63.2 years (9.1)<br><br>Gender:<br>• 10 females<br>• 61 males	Background and aim:<br>A training program (WiiNWalk) was shown to improve access to and effectiveness of rehabilitation for people with lower limb amputation. The program is based on social cognitive theory and involves all four sources of self-efficacy and was found to be feasible, safe, and effective for people with lower limb amputation. This exergaming intervention aimed to improve walking capacity and balance in older adults with lower limb amputation.<br><br>Devices/exergames used:<br>Participants used a Wii Fit balance board to play selected Wii Fit games, including yoga, balance games, strength training, and aerobics.<br><br>Intended protocol:<br>Participants were asked to play independently at home as much as they liked for 4 weeks. Before this unsupervised phase, participants first completed a supervised exergaming intervention: 1 week of in-person sessions at a rehabilitation facility followed by 3 weeks of a group training via teleconference at home.<br><br>Participant support:<br>• Training: in the supervised phase, participants were asked to play Wii Fit games for 40 min 3 times a week in the clinic (one week, participants played in groups of 3) and in their home (3 weeks, participants played individually, but could see each other playing and talk with each other via tablet devices). A trainer provided individualized guidance and feedback and encouraged group members to provide each other with feedback. In addition, participants spent 20-50 min observing and engaging with the group.	CG played cognitive digital games (Wii Big Brain Academy Degree), and solved cognitive tasks (identify, memorize, analyze, compute, and visualize)	Physical health:<br>• Walking capacity (2MWT)<br>• Dynamic standing balance (FSST)<br>• Lower limb functioning (SPPB)<br><br>Quality of life:<br>• Balance confidence (ABC)<br><br>Experiences:<br>• Adherence: percentage of sessions completed<br>• Adverse outcomes: falls between baseline and follow-up.<br><br>Physical health and quality of life were measured at baseline, after 4 weeks (supervised), after 9 weeks (unsupervised), and after 12 weeks (follow-up).	Physical health:<br>• No significant between-group differences in physical health.<br>• Walking capacity in the IG numerically increased in the supervised phase and decreased in the unsupervised phase, resulting in an overall increase at follow-up. Walking capacity in the CG at follow-up returned to baseline level.<br>• Lower limb functioning gradually increased in both groups and was higher in the IG (CG returned to baseline level).<br>• Dynamic standing balance gradually increased in both groups and was numerically better in the IG (at the follow-up, CG returned to the baseline level).<br><br>Quality of life:<br>• IG showed a higher balance confidence than the CG. There was a slight improvement in the IG, and a larger decrease in the CG.<br><br>Experiences:<br>• Adherence: good during the supervised phase (89% of supervised sessions), moderate to poor during the subsequent unsupervised phase (4.1 times over 4 weeks, i.e., only about 34% of the intended sessions).<br>• Adverse outcomes: one fall after the intervention period

Tefertiller et al. (2019)<br><br>United States	Parallel RCT (assessor-blinded)<br><br>Simple randomization	Traumatic brain injury (TBI) (criterion: 1-year post moderate to severe traumatic brain injury)	63 adults ≤ 65 years with ≥ 1 year post moderate to severe TBI.<br><br>Baseline and final/analyzed sample:<br>*n* = 63<br>(IG: 31;<br>CG: 32).<br><br>Mean age (standard deviation):<br>• IG: 48.1 years (12.4)<br>• CG: 49.5 years (12.4)<br><br>Gender:<br>• 24 females<br>• 39 males	Background and aim:<br>Compliance with traditional written home-based exercise programs has been poor. Using virtual reality was found to increase balance and balance confidence in people with neurologic conditions, while evidence regarding traumatic brain injury was limited. This exergaming intervention aimed to support home-based physical therapy to improve balance in people with traumatic brain injury.<br><br>Devices/exergames used:<br>Participants used an Xbox Kinect to play Kinect Adventures and Kinect Sports (20,000 leaks, soccer, table tennis, rallyball, beach volleyball, river rush). The games were determined based on the most impaired subscale of the BESTest, and exercise difficulty (basic, intermediate, and advanced) was determined by the total CB&M score.<br><br>Intended protocol:<br>Participants were asked to play independently at home for 30 min 3 to 4 times per week for 12 weeks. Difficulty was adjusted after 6 weeks.<br><br>Participant support:<br>• Setup: a physical therapist installed the gaming system.<br>• Training: the physical therapist trained participants and returned after one week to ensure participant understanding.	CG did traditional home-based exercise program.	Physical health:<br>• Balance (BESTest; CB&M)<br><br>Quality of life:<br>• Balance confidence (ABC)<br>• Community participation (PART-O)<br><br>Experiences:<br>• Adherence: Diary/activity log<br>• Adverse outcomes: separate log<br><br>Physical health and quality of life were measured at baseline, after 6 weeks, after 3 months, and after 6 months.	Physical health:<br>• Compared to baseline, both groups showed higher BESTest and CB&M scores after 6 and 12 weeks, and at 12 weeks follow-up (no significant between-group differences).<br><br>Quality of life:<br>• No significant within-group changes or between-group differences.<br><br>Experiences:<br>• Adherence was moderate: Adherence was similar between-groups but decreased within-group from about 4 to about 2 weekly sessions (3 to 4 weekly sessions were intended).<br>• Adverse outcomes: none

Thomas et al. (2017)<br><br>United Kingdom	Parallel pilot RCT (researcher not blinded, clinical scientist blinded)<br><br>Block randomization with varying block size in a 1:1 ratio	Multiple Sclerosis (MS) (clinically definite diagnosis)	30 adults who visited a MS Service in a secondary care setting.<br><br>Baseline sample:<br>*n* = 30<br>(IG: 15;<br>CG: 15).<br><br>Mean age (standard deviation):<br>• IG: 50.9 years (8.1)<br>• CG: 47.6 years (9.3)<br><br>Gender:<br>• 27 females<br>• 3 males<br><br>Final/analyzed sample:<br>*n* = 28<br>(IG: 13;<br>CG: 15).	Background and aim:<br>People with multiple sclerosis typically are less active than the general population, and exergaming could motivate for physical activity and increase their confidence in physical activity. This work aimed to examine the (cost-)effectiveness, adherence, and acceptance of a developed intervention. The intervention considered motivational interviewing, social cognitive theory, cognitive behavioral theory, self-determination theory, and behavior change techniques and aimed to support physical activity and well-being of people with multiple sclerosis at home.<br><br>Devices/exergames used:<br>Participants used a Wii console to play Wii Sports, and Wii Sports Resort, and a Wii Fit balance board to play Wii Fit Plus (exact games are not specified).<br><br>Intended protocol:<br>Participants completed an individualized training program independently at home for 12 months (IG) or 6 months (CG after crossover).<br><br>Participant support:<br>• Instructions: the exergaming intervention and its purpose were explained to participants by senior physiotherapists in the hospital and participants' home. Participants received a handbook, which was about benefits of physical activity and provided advice regarding how to stay physically active and how to use the Wii safely. In addition, participants received a personal activity workbook that facilitated individualized goal setting, feedback, action and coping planning and monitoring of progress.<br>• Training: the first two sessions were supervised in the hospital; the third session was supervised in patients' homes.<br>• Contact: participants received regular individualized support (face-to-face, telephone or email).	CG received usual care, but also the same intervention after a 6-month delay.	Physical health:<br>• Physical activity (GLTEQ, activPAL3 tri-axal accelerometer)<br>• Balance/gait/mobility (2MWT, Step Test, Steady Stance Test, i-TUG, Gait Stride-time Rhythmicity, Static Posturography)<br>• Hand dexterity/coordination (NHPT)<br><br>Quality of life:<br>• Self-efficacy (SCI-ESES, MSSE)<br>• Health status (EQ-5D-5L)<br>• Fatigue (FSI)<br>• Anxiety and depression (HADS)<br>• Physical and psychological impact of multiple sclerosis (MSIS-29)<br>• Physical and mental quality of life (SF-36 v.2 PCS)<br><br>Experiences:<br>• Interviews (acceptability of study processes from participants' and physiotherapists' point of view)<br>• Self-reported daily play log (e.g., ratings of enjoyment and fatigue)<br>• Adherence in terms of self-reported daily play log (frequency, intensity, duration, reasons for non-use)<br>• Adverse outcomes based on contact with care team, physical assessment hospital visits, from physiotherapists via face-to-face/telephone contacts with participants, via daily play logs and qualitative interviews.<br><br>Physical health and quality of life were measured at baseline, after 6 months, and after 12 months. Experiences were measured after 6 and 12 months.	Physical health:<br>• Descriptively and in terms of effect sizes, physical activity, balance, and gait measures were higher after 6 months of exergaming (however, confidence intervals were wide and spanned zero).<br><br>Quality of life:<br>• Descriptively and in terms of effect sizes, more self-efficacy and less self-reported hospital depression and hospital anxiety, and psychological impact of multiple sclerosis on day-to-day life after 6 months of exergaming (however, confidence intervals were wide and spanned zero).<br><br>Experiences:<br>• Adherence: Participants in the IG were exergaming around 2 days per week on average (no overall duration was intended).<br>• Adverse outcomes: Leg pain, overdoing it, aggravating existing scar tissue, back pain, discomfort in hand, aggravating existing knee injury, catching toe on the Balance Board, falls and near falls (none classified as serious).<br>• Most frequent reasons for not exercising were no time, too tired and feeling unwell.<br>• Qualitative results indicate that the intervention was well-received. Participants reported a wide range of benefits related to both physical and mental health (e.g., improved physical activity and confidence, better sleep and mood, and relief in symptoms such as pain and fatigue). Participants tended to exercise alone, in the morning or afternoon, with light-to-moderate intensity. Balance exercises, yoga and aerobics were the most common activities used.

Villumsen et al. (2019)<br><br>Denmark	Parallel RCT (assessor-blinded)<br><br>Block randomization with varying block size (block size: 8 to 10) in a 1:1 ratio	Prostate cancer (continuous androgen-deprivation therapy for ≥ 3 months prior to inclusion)	46 older men from regional hospitals receiving continuous androgen-deprivation therapy for ≥ 3 months.<br><br>Baseline sample:<br>*n* = 46<br>(IG: 23;<br>CG: 23).<br><br>Mean age (standard deviation):<br>• IG: 67.6 years (4.6)<br>• CG: 69.8 years (4.4)<br><br>Final/analyzed sample:<br>*n* = 41<br>(IG: 21;<br>CG: 20).	Background and aim:<br>People with prostate cancer usually receive androgen-deprivation therapy, which has been related to, i. a., loss of muscle and bone mass and decreased quality of life. However, aerobic and resistance exercises can counteract these adverse effects as shown in supervised contexts. This exergaming intervention aimed to explore the effects of home-based exergaming to support home-based rehabilitation for people with prostate cancer.<br><br>Devices/exergames used:<br>Participants used an Xbox 360 Kinect system to play the games Adventures, Sports, and Your Shape Fitness Evolved 2012 at their own convenience to complete aerobic and strength exercises, including a warm-up and cool down period. Participants used free weights to increase exercise intensity.<br><br>Intended protocol:<br>Participants were asked to play independently at home for 1 hour, 3 times a week for 12 weeks.<br><br>Participant support:<br>• Instructions: participants were individually instructed by a physiotherapist for 90 min.<br>• Contact: a research assistant contacted participants via phone fortnightly to ensure compliance and to assess adverse events and changes in medication.	CG continued their normal daily activities.	Physical health:<br>• Walking capacity (6MWT)<br>• Leg extensor power (power rig)<br>• Body composition (impedance analyzer)<br>• Physical activity level (Godin Leisure-time exercise questionnaire)<br><br>Quality of life:<br>• General quality of life (EORTC QLQ-C30, FACT-P)<br>• Fatigue (FACT-F)<br><br>Experiences:<br>• Exercise diary<br>• Fortnightly phone calls to ensure compliance and to assess adverse events.	Physical health:<br>• Only the IG improved significantly in walking capacity.<br><br>Quality of life:<br>• No significant changes, but global health status increased numerically in the IG more than in the CG.<br><br>Experiences:<br>• Adherence was very good: Participants reported to have exercised 153.5 min/week (85.28% of the instructed time; 3 h per week were intended).<br>• Adverse outcomes: One participant in the IG discontinued because of severe non-heart-related chest pain due to surgical clips in the thorax.

Yacoby et al. (2019)<br><br>Israel	Parallel pilot RCT (assessor-blinded)<br><br>Block randomization in a 1:1 ratio	Stroke (onset of stroke within 6–36 months, clinical assessment and a positive computed tomography or magnetic resonance imaging scan)	24 adults aged ≤ 80 years<br><br>Baseline sample:<br>*n* = 24<br>(IG: 13;<br>CG: 11).<br><br>Mean age (standard deviation):<br>• IG: 59.1 years (10.5)<br>• CG: 64.9 years (6.9)<br><br>Final/analyzed sample:<br>*n* = 24<br>(IG: 13;<br>CG: 11).<br><br>Gender:<br>• 9 females<br>• 15 males	Background and aim:<br>Exergames are thought to benefit neuroplasticity and to motivate people who have had stroke to self-training of their weaker upper extremity. Prior studies showed the effectiveness of home-based rehabilitation systems, which are yet less available, affordable, and user-friendly compared to commercial exergames. This exergaming intervention aimed to support home-based hand rehabilitation for people who have had stroke.<br><br>Devices/exergames used:<br>Participants who were safe to played while standing used an Xbox Kinect system, other participants used a PlayStation EyeToy while seated to play 3-5 games at their own convenience to complete arm exercises.<br><br>Intended protocol:<br>Participants were asked to play independently at home for one hour 6 times a week for 5 weeks (4 additional weeks were optional). Participants were encouraged to play with their weaker arm.<br><br>Participant support:<br>• Setup: an occupational therapist installed the gaming system at home an arranged a safe playing area.<br>• Training: the occupational therapist taught each participant how to perform the self-training.<br>• Contact: daily contact during the first week (phone or text message), weekly contact during weeks 2–5. Home visit after 2 weeks to ensure that the training was conducted correctly. Participants should contact the therapist in case of adverse effects or technical problems.	CG did arm exercises based on the Graded Repetitive Arm Supplementary Program (GRASP), and additional lower extremity exercises (stretching, marching, and stepping).	Physical health:<br>• Perceived upper extremity improvement (5-point scale)<br>• Perceived balance improvement (5-point scale)<br><br>Quality of life:<br>• NI<br><br>Experiences:<br>• Adherence in terms of self-reported daily play log (duration)<br>• Adverse outcomes based on contact with care team and via daily play logs.<br>• Daily perceived enjoyment (5-point scale)<br>• Overall satisfaction (5-point scale)<br>• Exercise-related dyspnea (Borg CR-10 scale)<br>• Family assistance needed (3-point scale)	Physical health:<br>• Perceived upper extremity improvement was more frequent in the CG (63% vs. 38%), perceived balance improvement was more frequent in the IG (69% vs. 27%).<br><br>Quality of life:<br>• NI<br><br>Experiences:<br>• Adherence: 11 of 13 (85 %, IG) and 9 of 11 (82 %, CG) participants completed the intervention. Participants in the IG played 18.8 h in 5 weeks on average (of 30 h intended, 62.67%). Participants in the CG played 27.4 h in 5 weeks on average (of 30 h intended, 91.33%).<br>• Adverse outcomes: none<br>• Perceived enjoyment was non-significantly higher in the IG.<br>• Overall satisfaction was moderate to high in both groups.<br>• Perceived exertion was light in both groups.<br>• Most participants rated family assistance needed as low in the IG (61%) and CG (54%).

Yuen et al. (2019)<br><br>United States	Parallel pilot RCT (assessor-blinded)<br><br>Block randomization (block size: 4) in a 1:1 ratio	Idiopathic pulmonary fibrosis (2011 guidelines of the American Thoracic Society, the European Respiratory Society, the Japanese Respiratory Society, and the Latin American Thoracic Associa-tion)	20 adults aged ≥ 40 from an interstitial lung disease clinic<br><br>Baseline and final/analyzed sample:<br>*n* = 20<br>(IG: 10;<br>CG: 10).<br><br>Mean age (standard deviation):<br>• IG: 67.4 years (7.4)<br>• CG: 72.2 years (8.4)<br><br>Gender:<br>• 7 females<br>• 13 males	Background and aim:<br>The use of exergames at home was shown to increase functional performance and quality of life of people with chronic obstructive pulmonary disease. However, experimental evidence is lacking on the feasibility and effectiveness of using exergames at home for people with idiopathic pulmonary fibrosis. This exergaming intervention aimed to support home-based rehabilitation for people with idiopathic pulmonary fibrosis.<br><br>Devices/exergames used:<br>Participants used a Wii U balance board to play Wii Fit games (exact games are not specified).<br><br>Intended protocol:<br>Participants were asked to play independently at home for 30 min 3 times a week for 12 weeks. Participants should play at an intensity corresponding to a perceived dyspnea level of 3 to 5 (moderate to heavy). In addition, participants were encouraged to engage in physical activity for 30 min 3 times a week.<br><br>Participant support:<br>• Contact: one week after baseline, participants received a phone call to check for technical problems; participants also received monthly phone calls with the aim to remind and encourage participants to follow the protocol.	CG played a cognitive digital game on Wii U. In addition, participants were encouraged to engage in physical activity for 30 min 3 times a week.	Physical health:<br>• Walking capacity (6MWT)<br><br>Quality of life:<br>• St George's Respiratory Questionnaire (SGRQ)<br><br>Experiences:<br>• Adherence: Participants estimated frequency and duration in the post-study survey<br>• Adverse outcomes: measurement approach unknown<br>• Exercise-related dyspnea (Borg CR-10 scale)<br><br>Physical health and quality of life were measured at baseline and after 3 months.	Physical health:<br>• No significant improvement in walking capacity.<br><br>Quality of life:<br>• No significant improvement in health-related quality of life.<br><br>Experiences:<br>• Adherence was poor: On average, 20% (*SD* = 23) for completion of prescribed sessions, 39% (34) for the prescribed frequency, and 42% (36) for the prescribed duration (36 sessions and 18 h in 12 weeks were intended).<br>• Adverse outcomes: none<br>• Exergaming group showed higher exercise-related dyspnea.

Zadro et al. (2019)<br><br>Secondary analysis: Zadro et al. (2020)<br><br>Australia	Parallel RCT (assessor-blinded)<br><br>Block randomization (block size: 6) in a 1:1 ratio	Chronic low back pain (nonspecific mechanical low back pain for ≥ 3 months, pain intensity of ≥ 3 out of 10 on a numeric rating scale)	60 adults aged > 55 with low back pain since ≥ 3 months<br><br>Baseline sample:<br>*n* = 60<br>(IG: 30;<br>CG: 30).<br><br>Mean age (standard deviation):<br>• IG: 68.8 years (5.5)<br>• CG: 67.8 years (6.0)<br><br>Gender:<br>• 31 females<br>• 29 males<br><br>Final/analyzed sample:<br>*n* = 57<br>(IG: 29;<br>CG: 28).	Background and aim:<br>Structured exercising can reduce pain and increase function in older people with chronic lower back pain. Exergames could motivate older people to self-manage their chronic lower back pain in terms of improving pain self-efficacy and reducing care-seeking. This home-based exergaming intervention aimed to support self-management in terms of pain self-efficacy and care-seeking for older people with chronic low back pain.<br><br>Devices/exergames used:<br>Participants used a Wii U to play Wii Fit U games (yoga, muscle/strength training, aerobics, and balance games). Games that required movements in which patients were unsafe or that increased their pain at least by 20 % were removed from the list. Participants could adjust exercise intensity in terms of progression to maintain a moderate level of perceived exertion or in terms of reduction to maintain a similar perceived exertion.<br><br>Intended protocol:<br>Participants were asked to play independently at home for 60 min 3 times a week (with at least one day in between) for 8 weeks.<br><br>Participant support:<br>• Setup: a physical therapist with 3 years clinical experiments set up the game device and guided participants through the first session (1–2 h).<br>• Instructions: participants received a booklet outlining a range of flexibility, body weight resistance, and aerobic exercises preselected by the research team to standardize the intervention. Participants were encouraged to modify exercises they found too difficult.<br>• Contact: fortnightly calls from a physical therapist to encourage participants and to assess adverse events or technical issues.	CG continued usual activities.	Physical health:<br>• Function (PSFS)<br>• Engagement in physical activity (RAPA)<br><br>Quality of life:<br>• Pain self-efficacy (PSEQ)<br>• Care-seeking (3-item questionnaire)<br>• Falls efficacy (FES-I)<br>• Pain intensity over the last week<br>• Disability (RMDQ)<br>• Fear of movement/reinjury (TSK)<br><br>Experiences:<br>• 12-item questionnaire (usability, exercise variation, ease of exercise progression, extent to which symptoms interfered with the program, overall experience)<br>• Adherence: exercise diary<br>• Adverse outcomes: telephone calls<br><br>Pain self-efficacy, engagement in physical activity levels, and care-seeking were measured at baseline, after 2 months, after 3 months, and after 6 months. Function, falls efficacy, pain intensity, disability, fear of movement/reinjury were measured at baseline and after 8 weeks. Experiences were measured at different time points.	Physical health:<br>• IG showed greater improvement in function at post-intervention, but not if they had a family history of activity-limiting lower back pain.<br>• IG was significantly more likely to engage in flexibility exercises at least once per week after 6 months.<br>• IG was more active in the long term (after 6 months) and showed better function when family members were also engaging in moderate or vigorous-intensity physical activity.<br><br>Quality of life:<br>• IG showed higher pain self-efficacy after 6 months (but not at post-intervention and after 3 months).<br>• IG reported significantly lower pain intensity over the last week.<br><br>Experiences:<br>• Adherence was good: on average, participants completed 70.8% of the total recommended exercise time, and 85.1% of the total number of recommended sessions. However, median values indicate lower adherence (24 h and 24 sessions in 8 weeks were intended).<br>• Adverse outcomes: none related to exergaming but exercising symptoms following a session.<br>• IG reported high usability, exercise variety, and challenge.

Zahedian-Nasab et al. (2021)<br><br>Iran	Parallel RCT (blinding unknown)<br><br>Double randomized block permutation (block size: 4)	Fall risk (Timed Up and Go score of 14–20)	60 adults living in nursing homes.<br><br>Baseline and final/analyzed sample:<br>*n* = 60<br>(IG: 30;<br>CG: 30).<br><br>Mean age (standard deviation):<br>• IG: 69.67 years (7.73)<br>• CG: 72 years (7.81)<br><br>Gender:<br>• 16 females<br>• 44 males	Background and aim:<br>Older people have a higher fall risk and fear of falling, which can be reduced by improving physical performance. Exergaming was found to improve balance in older people, but previous works were about specific pathologies or limited regarding sample size and intervention duration. This intervention aimed to support rehabilitation of fall risk in terms of balance and fear of falling for older people living in nursing homes.<br><br>Devices/exergames used:<br>Participants used an Xbox Kinect to play the Xbox Kinect sports pack (ski, penalty, goalkeeper, and darts). Games were preselected and required the application of upper and lower organs while standing.<br><br>Intended protocol:<br>Participants were asked to play for 30-60 min twice a week for 6 weeks. Participants should exercise on special mats to prevent injury. Exergaming was stopped in case of fatigue, pain, or dyspnea.<br><br>Participant support:<br>• Instructions: exergames were explained to participants.<br>• Contact: participants were encouraged by the researcher during the game.	CG received routine programs of the nursing homes.	Physical health:<br>• Balance (BBS, TUG)<br><br>Quality of life:<br>• Fear of falling (FES)<br><br>Experiences:<br>• NI	Physical health:<br>• Significant increase in balance in IG, but not in CG.<br><br>Quality of life:<br>• Significant decrease in fear of falling in IG, but not in CG.<br><br>Experiences:<br>• NI

Zondervan et al. (2016)<br><br>United States	Crossover RCT (assessor-blinded)<br><br>Adaptive randomization	Chronic stroke (last stroke > 6 months prior to the study)	18 adults<br><br>Baseline sample:<br>*n* = 18<br>(IG: 9;<br>CG: 9).<br><br>Final/analyzed sample:<br>*n* = 17<br>(IG: 9;<br>CG: 8).<br><br>Mean age (range):<br>• IG: 60 years (45–74)<br>• CG: 59 years (35–74)<br><br>Gender:<br>• 7 females<br>• 10 males	Background and aim:<br>Hand impairments are common in people who have had stroke, yet hand exercises are not performed to the degree required for recovery due to missing motivation, compliance, or access and costs related to therapy. A glove (MusicGlove) was developed that allows for self-guided hand exercises with a digital game, yet its feasibility and effectiveness need to be examined. This exergaming intervention aimed to support home-based hand rehabilitation for people after chronic stroke.<br><br>Devices/exergames used:<br>Participants used a MusicGlove device and a laptop with the software preinstalled.<br><br>Intended protocol:<br>Participants were asked to play for at least 3 h per week over at least 3 sessions per week, for 3 consecutive weeks (9 h in total).<br><br>Participant support:<br>• Setup: the exergame was preinstalled on a laptop.<br>• Training: participants were introduced to the hardware and preinstalled software (15 min).<br>• Contact: a research therapist or nurse contacted participants at least once a week to ask about technical difficulties and adverse effects.	CG received a booklet of tabletop exercises for conventional home therapy of the hand.	Physical health:<br>• Gripping function (BBT)<br>• Motor activity log<br>• Hand dexterity/coordination (NHPT)<br>• Arm function (ARAT)<br><br>Quality of life:<br>• NI<br><br>Experiences:<br>• Written exercise log	Physical health:<br>• Both groups significantly increased their gripping function.<br>• Only the IG significantly improved motor activity (quality of movement and amount of use).<br><br>Quality of life:<br>• NI<br><br>Experiences:<br>• Self-reported adherence was good in both groups, but the CG continued to exercise after the intended exercise period of 3 weeks (until follow-up). Participants in the IG used MusicGlove more frequently after the first week, but this difference was not significant.<br>• Adverse outcomes: NI

CG, Control/Comparison Group; IG, Intervention Group; NI, No Information; RCT, Randomized Controlled Trial.

2MWT, 2-Minute Walk Test; 6MWT, 6-Minute Walk Test; 25-FWT, 25-Foot Walking Test; ABC, Activities Balance Confidence Questionnaire; ARAT, Action Research Arm Test; BBS, Berg Balance Scale; BBT, Box and Blocks Test; BESTest, Balance Evaluation Systems Test; CB&M, Community Balance and Mobility Scale; COPM, Canadian Occupational Performance Measure; DAS, Disease Activity Score; DEXA: Dual-Energy X-ray Absorptiometry; DGI, Dynamic Gait Index; EQ-5D-3L, EuroQol-5 Dimensions-3 Levels; EQ-5D-5L, EuroQol-5 Dimensions-5 Levels; FAAM, Foot and Ankle Ability Measure; FACIT, Functional Assessment of Chronic Illness Therapy; FES, Falls Efficacy Scale; FES-I, Falls Efficacy Scale-International; FSI, Fatigue Symptom Inventory; FSST, 4-Step Square Test; GLTEQ, Godin Leisure-Time Exercise Questionnaire; GRASSP: Graded Redefined Assessment of Strength, Sensation and Prehension; HADS, Hospital Anxiety and Depression Scale; HAQ, Health Assessment Questionnaire; i-TUG, Instrumented Timed Up and Go Test; LCI-5, Locomotor Capabilities Index in Amputees; MRS, Modified Rankin Scale; MSIS-29, 29-item Multiple Sclerosis Impact Scale; MSSE, Multiple Sclerosis Self-Efficacy Scale; NHPT, Nine-Hole Peg Test; PASE, Physical Activity for the Elderly; PSEQ, Pain Self-Efficacy Questionnaire; PSFS, Patient Specific Functional Scale; RAPA, Rapid Assessment of Physical Activity Questionnaire; RMDQ, Roland Morris Disability Questionnaire; SCI-ESES, Spinal Cord Injury Exercise Self-Efficacy Scale; SF-36 v.2 PCS, Medical Outcomes Short-Form Survey Version 2 Physical Component Summary; SGRQ, St George's Respiratory Questionnaire; SIS, Stroke Impact Scale Version 2.0; SOT, Sensory Organization Test; SPPB, Short Physical Performance Battery; TSK, Tampa Scale of Kinesiophobia; TUG, Timed Up and Go Test; VRBQ, Vestibular Rehabilitation Benefits Questionnaire; WWT, Walking While Talking Test.

Table S3. Training principles in the intervention and control/comparison groups.

Study	Variation	Specificity	Overload	Progression	Reversibility	Periodization and programming
Adie et al. (2017)	NI	IG: NI<br><br>CG: tailored arm exercises	NI	NI	NI	NI
Ambrosino et al. (2020)	IG: game changed after 10 min of play	IG: games were preselected (group level)	IG: NI	IG: NI	IG: NI	IG: game changed after 10 min of play (programming).
	CG: N/A	CG: N/A	CG: N/A	CG: N/A	CG: N/A	CG: N/A
Golla et al. (2018)	NI	IG: games were preselected (group level).<br><br>CG: 1 of 3 instructions based on physical abilities (individual level).	NI	NI	NI	NI
Imam et al. (2017)	IG: NI	IG: games were preselected (group level).	IG: NI	IG: independent progression to advanced levels	IG: NI	IG: NI
	CG: N/A	CG: N/A	CG: N/A	CG: N/A	CG: N/A	CG: N/A
Jaarsma et al. (2021)	IG: NI	IG: exercise duration could be shortened based on individual physical condition.	IG: NI	IG: in case of lower physical condition, exercise duration should be increased up to 30 min.	IG: NI	IG: NI
	CG: N/A	CG: N/A	CG: N/A	CG: N/A	CG: N/A	CG: N/A
Meldrum et al. (2015)	Weekly modified exercises	IG: balance board was modified from a stable to an unstable surface (group level).<br><br>CG: N/A	NI	Progressive increase	NI	Weekly modified exercises (non-linear periodization).

Prosperini et al. (2013)	IG: game changed after level progress or after 10 min of play.	IG: games allowed in the first 4 weeks were preselected (group level).	IG: NI	IG: independent progression to advanced levels.	IG: NI	IG: game changed after level progress or after 10 min of play (programming).
	CG: N/A	CG: N/A	CG: N/A	CG: N/A	CG: N/A	CG: N/A
Punt et al. (2016)	IG: NI	IG: games were preselected (group level), preferred difficulty level (individual level).	IG: NI	IG: exercise difficulty could be independently adjusted (no maximum predefined).	IG: NI	IG: NI
	CG1: additional exercises in consolidation phase (after 60 days).	CG1: exercise difficulty was based on progress (individual level).	CG1: NI	CG1: therapist adjusted exercise difficulty based on participants' progress.	CG1: NI	CG1: NI
	CG2: N/A	CG2: N/A	CG2: N/A	CG2: N/A	CG2: N/A	CG2: N/A
Sajid et al. (2016)	IG: NI	IG: games were preselected (individual level), initial intensity was determined (individual level).	IG: NI	IG: independent progression to advanced levels.	IG: NI	IG: gradual increase in exercise intensity (linear periodization).
	CG1: NI	CG1: initial intensity was determined (individual level).	CG1: NI	CG1: independent progression in terms of changing resistance bands and number of sets and sessions; in addition, encouragement for 5-20% weekly increase in step count (to reach 10,000 steps).	CG1: NI	CG1: gradual increase in exercise intensity (linear periodization).
	CG2: N/A	CG2: N/A	CG2: N/A	CG2: N/A	CG2: N/A	CG2: N/A
Sanders et al. (2020)	IG: no systematic manipulation (free choice).	IG: free choice between 3 difficulty levels and two game modes (individual level).	NI	IG: progression was optional but possible via change in difficulty and game mode.	NI	IG: NI
	CG: varying number of exercise repetitions.	CG: exercises, durations, and number of repetitions were prespecified in a booklet (group level).		CG: NI		CG: durations and number of repetitions were prespecified in a booklet (programming).

Sanders et al. (2022)	IG: no systematic manipulation (free choice).<br><br>CG: no systematic manipulation (exercise booklet).	IG: free choice between 3 difficulty levels and two game modes (individual level).<br><br>CG: exercises, durations, and number of repetitions were prespecified in a booklet (group level).	NI	IG: progression was optional but possible via change in difficulty and game mode.<br><br>CG: NI	NI	IG: NI<br><br>CG: number of repetitions were prespecified in a booklet (programming).
Tao et al. (2022)	IG and CG1: no systematic manipulation (free choice)<br><br>CG1: no systematic manipulation (free choice)<br><br>CG2: N/A	IG: NI<br><br>CG1: NI<br><br>CG2: N/A	IG: NI<br><br>CG1: NI<br><br>CG2: N/A	IG: NI<br><br>CG1: NI<br><br>CG2: N/A	IG: NI<br><br>CG1: NI<br><br>CG2: N/A	IG: NI<br><br>CG1: NI<br><br>CG2: N/A
Tefertiller et al. (2019)	NI	IG: games were preselected (individual level), exercise difficulty was predetermined (individual level).<br><br>CG: exercise difficulty was predetermined (individual level)	NI	Progressive increase by adjusting the difficulty halfway through the intervention.	NI	One-time adjustment of difficulty halfway through the intervention.
Thomas et al. (2017)	IG: modifications possible after review visits with the physiotherapist.<br><br>CG: N/A	IG: tailored exercise program, regular one-to-one support from a physiotherapist, personal activity workbook (all individual level).<br><br>CG: N/A	IG: NI<br><br>CG: N/A	IG: progression possible after review visits with the physiotherapist.<br><br>CG: N/A	IG: NI<br><br>CG: N/A	IG: adjustments via review visits with the physiotherapist.<br><br>CG: N/A
Villumsen et al. (2019)	IG: NI<br><br>CG: N/A	IG: 3 games were preselected (group level).<br><br>CG: N/A	IG: NI<br><br>CG: N/A	IG: progressive increase at own convenience using free weights of 0.5, 1.0 and 2.0 kg.<br><br>CG: N/A	IG: NI<br><br>CG: N/A	IG: gradual increase in exercise intensity (linear periodization).<br><br>CG: N/A

Yacoby et al. (2019)	NI	IG: 3–5 games were preselected (individual level), games were performed standing or seated (individual level).<br><br>CG: unclear (instruction manuals existed for 3 different levels of exercises).	NI	NI	NI	NI
Yuen et al. (2019)	IG: NI<br><br>CG: N/A	IG: NI<br><br>CG: N/A	IG: NI<br><br>CG: N/A	IG: NI<br><br>CG: N/A	IG: NI<br><br>CG: N/A	IG: NI<br><br>CG: N/A
Zadro et al. (2019)	IG: NI<br><br>CG: N/A	IG: games were preselected (group level), games that were unsafe or caused pain were removed (individual level).<br><br>CG: N/A	IG: NI<br><br>CG: N/A	IG: independent progression (increase or reduction) to maintain a similar perceived exertion and to balance difficulty.<br><br>CG: N/A	IG: NI<br><br>CG: N/A	IG: NI<br><br>CG: N/A
Zahedian-Nasab et al. (2021)	NI	IG: games were preselected (group level).<br><br>CG: NI	NI	NI	NI	NI
Zondervan et al. (2016)	IG: no systematic manipulation (free choice).<br><br>CG: no systematic manipulation (exercise booklet).	IG: NI<br><br>CG: exercises, durations, and number of repetitions were prespecified in a booklet (group level).	NI	NI	NI	NI

CG, Control/Comparison Group; IG, Intervention Group; N/A, Not Applicable; NI, No Information. For studies that include supervised and unsupervised uses of exergames, data only refer to the unsupervised phase.
